# Discriminating External and Internal Causes for Heading Changes in Freely Flying *Drosophila*

**DOI:** 10.1371/journal.pcbi.1002891

**Published:** 2013-02-28

**Authors:** Andrea Censi, Andrew D. Straw, Rosalyn W. Sayaman, Richard M. Murray, Michael H. Dickinson

**Affiliations:** 1 Control & Dynamical Systems, California Institute of Technology, Pasadena, California, United States of America; 2 Research Institute of Molecular Pathology (IMP), Vienna, Austria; 3 Division of Biology, California Institute of Technology, Pasadena, California, United States of America; 4 Department of Biology, University of Washington, Seattle, Washington, United States of America; Indiana University, United States of America

## Abstract

As animals move through the world in search of resources, they change course in reaction to both external sensory cues and internally-generated programs. Elucidating the functional logic of complex search algorithms is challenging because the observable actions of the animal cannot be unambiguously assigned to externally- or internally-triggered events. We present a technique that addresses this challenge by assessing quantitatively the contribution of external stimuli and internal processes. We apply this technique to the analysis of rapid turns (“saccades”) of freely flying *Drosophila melanogaster*. We show that a single scalar feature computed from the visual stimulus experienced by the animal is sufficient to explain a majority (93%) of the turning decisions. We automatically estimate this scalar value from the observable trajectory, without any assumption regarding the sensory processing. A posteriori, we show that the estimated feature field is consistent with previous results measured in other experimental conditions. The remaining turning decisions, not explained by this feature of the visual input, may be attributed to a combination of deterministic processes based on unobservable internal states and purely stochastic behavior. We cannot distinguish these contributions using external observations alone, but we are able to provide a quantitative bound of their relative importance with respect to stimulus-triggered decisions. Our results suggest that comparatively few saccades in free-flying conditions are a result of an intrinsic spontaneous process, contrary to previous suggestions. We discuss how this technique could be generalized for use in other systems and employed as a tool for classifying effects into sensory, decision, and motor categories when used to analyze data from genetic behavioral screens.

## Author Summary

Researchers have spent considerable effort studying how specific sensory stimuli elicit behavioral responses and how other behaviors may arise independent of external inputs in conditions of sensory deprivation. Yet an animal in its natural context, such as searching for food or mates, turns both in response to external stimuli and intrinsic, possibly stochastic, decisions. We show how to estimate the contribution of vision and internal causes on the observable behavior of freely flying Drosophila. We developed a dimensionality reduction scheme that finds a one-dimensional feature of the visual stimulus that best predicts turning decisions. This visual feature extraction is consistent with previous literature on visually elicited fly turning and predicts a large majority of turns in the tested environment. The rarity of stimulus-independent events suggests that fly behavior is more deterministic than previously suggested and that, more generally, animal search strategies may be dominated by responses to stimuli with only modest contributions from internal causes.

## Introduction

Active movement is one of the defining features of animals, and the use of locomotion to search for resources within the environment is likely among the most ancient of behaviors. Observations on motile organisms, ranging in scale from bacteria to whales, indicate that search patterns are structured by a combination of internal processes and external cues [1], [2]. Sensory systems enable organisms to detect favorable objects at a great distance [3]–[5] and they use this ability to localize resources by either directed motion (taxis) or changes in locomotor statistics (kinesis). Prior research suggests that, in the absence of external cues, the animal behavior is generated by internal processes, and that the overall animal fitness is sensitive to the exact characteristics of this internal process (e.g., Levy statistics) [6]–[18]; it has also been questioned whether observed large-scale statistics can give any insight on an internal process that generated the behavior, and whether the internal processes can dominate over stimuli-elicited behavior [19]–[22]. As for the internal processes, these can be divided into truly stochastic sources, and deterministic results of a deliberate, but unobservable, internal mechanism based on internal metabolic/neural states. When observing an intact motile organism, it is not easy to determine which components of its locomotion behavior are triggered by internal processes versus external cues, yet such classification is essential for deciphering the underlying logic of its movement and search behavior. The task is further complicated by the fact that an external observer might not be able to distinguish between truly stochastic processes and the deterministic results of a deliberate, but unobservable, internal mechanism. For example, software pseudo-random number generators produce strictly deterministic sequences, which appear to be random to an external observer who does not have access to the internal state of the system [23]. A major goal of both cell biology and neuroscience is explaining the molecular and cellular bases of these three qualitatively different processes (sensory-driven, purely stochastic, and deterministically based on internal states).

If the salient features of the external world are known, it is possible to gain insight into sensory-driven behaviors through the use of sensory-response correlation [24]. The analysis of the internally-driven processes is much more challenging. Given uncertainty in measurement and the inability to perfectly reproduce experimental conditions from trial to trial, variability in the results of behavioral experiments has often been treated as a limit on our ability to measure stimulus-driven behavior. In this view, variability in responses from trial to trial reflects irrelevant components of behavior, which are averaged until the mean—interpreted as the response the animal ideally would have produced—becomes clear [25]. From the opposite perspective, many researchers have attempted to artificially remove all relevant sensory input to an animal and measure behaviors in conditions of sensory deprivation to reveal intrinsic properties, especially the statistical distributions of behaviors [26]–[28]. Although focusing in isolation on either the stochastic [9], [29], [26] or the sensory components [30] of search behavior have provided key insights, neither of these extremes is sufficient to capture the full range of processes at play as an animal moves under natural conditions. Attempts to investigate the interaction of internal and external processes include studies of bacteria [31] and nematode worms [32], [33], organisms for whom chemicals provide the most salient cues for food search. For larger animals with image-forming eyes, vision may provide another essential cue in search algorithms, because vision is the only sense which allows to perceive remote parts of the environment. Often vision cannot be considered separately from the mechanics of locomotion [34].

Flies are a model of computational efficiency and robustness, to date not equaled by artificial systems, which often seek to imitate nature [35], [36]. Much is known about fly vision [37], [38]. Since the pioneering work of Kennedy [39] and Mittelstadt [40], the behavioral responses of flies to experimenter-defined visual stimuli have been extensively investigated. Electrophysiological recordings have complemented and extended our knowledge of phenomena such as the neural basis of motion detection [41]–[44] and other key aspects of sensory processing, such as receptive field tuning [45]. However, there are many challenges in the identification of neural processing and how it produces complex behavior, especially as regards the characterization of “discrete” behaviors, such as the rapid turns (“saccades”) of *Drosophila*, which are the object of this study. In fact, many studies which offered complete characterization of the animal response are limited to “continuous” behavior, for which they provide linear (or “linearized”) models [46]–[49]; this allows using techniques such as linear system identification.

Identifying the neural causes for “discrete” behavior involves solving a different set of problems. Firstly, there are the problems of segmentation and classification of behaviors (including the definition of what “behavior” and “a behavior” are), for which it is often necessary the use of nonlinear machine learning methods [50]. Then, there is the problem of building models that can correlate the stimulus with the behavior(s). While it is possible to postulate models that also integrate well with our understanding of lower-level behavior [51], [52], it is not clear how such methods can be identified from the data. On the practical side, it is evident that discrete decisions, such as turning decisions, are meant to guide exploration and therefore should be investigated in naturalistic situations. This poses practical problems of tracking the animal position in a large environment, and it also precludes (at the current level of technology) the uses of direct neural recording. In fact, comparatively few attempts have been made to correlate parameters of visual stimulus with behavioral responses in unrestrained conditions [53]–[56].

In this work, we present an analysis that can quantitatively discriminate the effect of visual stimulus as opposed to internal processes in the generation of saccades in the fruit fly. Our conclusions are that visual stimulus has a dominant role. One important message of our work is that it is very difficult to identify models of complex behavior that can explain everything, often because insufficient data can be collected. Therefore, it is important to “search for simplicity” [57], for example by framing the problem as dimensionality reduction, and to use models that a posteriori can justify their assumptions. While we describe this analysis for visual processing in Drosophila, our goal is to construct a general method that can be used for other sensory systems, other animal species, or in the context of genetic screens.

## Methods

### Fly care and experimental treatment

Flies from the laboratory stock derived from 200 wild-caught females were reared on a 16 h:8 h light dark cycle under standard laboratory conditions. Three day old adult female flies were anesthetized with cold and individually housed within centrifuge tubes containing a moist tissue paper. Flies were starved (but provided with water) in the tubes for four to six hours before being released into the flight arena. Most flies would immediately begin flying, and we terminated tracking after the fly landed. We then removed each fly with a wand attachment of a vacuum cleaner before introducing another fly. Thus, each recorded trajectory is derived from a fly's initial experience exploring the novel environment.

**Figure 1:**
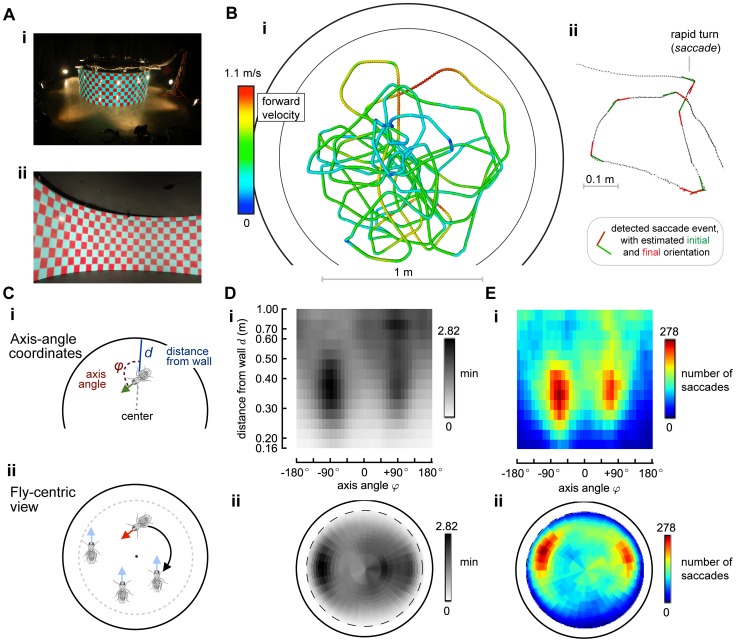
Data collection, saccade detection, reduced coordinate space, time histogram, number of saccades histogram. Panel A shows the experimental setup: the fly is tracked in a circular arena of 1 m radius. The retro-illuminated checkerboard pattern gives a uniform stimulus to the fly. Panel B-i shows some of the trajectories recorded in the arena. The trajectory can be interpreted as a mix of smooth turns and rapid turns, called “saccades”, which are responsible for most of the total angular displacement of the animal. We wrote software to detect these saccades events, based on two different algorithms, documented in the Supplemental Materials. In this paper, we only consider these discrete saccade events (Panel B-ii). Panel C shows the two coordinate systems we use in this paper. We take advantage of the circular symmetry of the environment, along with a hypothesis of planarity, to reduce the degrees of freedom to 2. Panel C-i shows the choice of the axis-angle/distance from the wall coordinates. Panel C-ii shows the “fly-centric view”. The fly configuration is reduced to 2 spatial coordinates by rotating the configuration so that the animal points “up” with respect to the diagram. We remark that all the results in this paper do not depend on the choice of coordinates. Panel D shows a density plot of 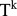, which is the time spent at each configuration 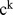. Panel E shows the number of saccades (both left and right) detected at each configuration.

The flight arena was a 2 meter diameter, 80 cm high cylinder (see Figure 1A). 10 cm×10 cm red and green gel filters (Roscolux) were attached to the arena in a regular checkerboard arrangement and provided a high contrast visual stimulus to flies near the wall. One meter from the wall (i.e., at the center of the arena), the angular wavelength of this pattern was ∼11°, and consequently would be twice the inter-ommatidial spacing of a ∼5.5° in *Drosophila* [58]. The particular red and green filters were chosen to have similar infrared transmission to facilitate tracking using cameras outfitted with long (IR) pass filters. The arena was illuminated from outside with a circular array of eight 750W Fresnel stage lights pointing towards the arena center. These lights provided both visible and infrared light for fly visual responses and machine vision tracking, respectively.

### Fly tracking

A detailed description of our tracking system may be found in [59]. Briefly, we used 11 cameras (6 monochrome Pt. Grey Firefly MV USB cameras and 5 monochrome Basler A602f cameras) with wide-angle lenses and infrared pass, visible cut filters (R72, Hoya Filters) to view the interior volume of the flight chamber. The cameras were positioned so that a fly within the tracking volume was viewed by 2 or more cameras at any given time, enabling a 3D estimate of its position (Figure 1Bi). The cameras were first calibrated to compensate for image warping non-linearities (deviations from the pinhole model) and then the extrinsic and intrinsic parameters describing the pinhole model were found. Flies were tracked with an extended Kalman filter (EKF), in which the motion model was a linear constant velocity model, and fly maneuvering is captured by the stochastic component of the Kalman filter. Because tracking updates occurred at a high rate (60 fps) relative to fly maneuvering, we found this simplification to work well in practice. The 3D estimate of the fly position is recovered by triangulation from the 2D tracking data of each camera, and taking into account the relative uncertainty of each observations.

### Saccade detection

Many species of flies, including *Drosophila*, exhibit rapid changes in heading as they fly, termed “saccades” [53]. Between saccades, flies tend to maintain an approximately straight course, and saccades account for at least 80% of the total net change in heading during flight [60]. There is little doubt that saccades can be triggered by visual stimuli, but the degree to which visual feedback plays a role in determining the velocity, duration, and amplitude of the resulting turn is unclear. Experiments using a magnetic tether, which permits free rotation about the yaw axis, suggest that flies do not respond to visual feedback during a saccade [61]. On the other hand, Stewart *et al.* [56] have observed a rebound effect after saccades in free flight, which they suggest is consistent with active optomotor feedback during the maneuver. This discrepancy is not of direct interest here, however, as we deal exclusively with the decision of *initiating* a saccade.

To analyze saccades within a flight trajectory, one should choose a detection algorithm that, given the trajectory data, returns a series of saccade events, possibly with other attributes such as direction, amplitude, velocity, etc. In the past, several detection algorithms have been proposed, each one implicitly using a slightly different definition of saccade, and each one able to compensate for different sources of noise. In practice, large saccades are such distinct events that all algorithms agree with respect to most classifications, but different algorithms may disagree on detection of small saccades. We make sure that our results are robust to the choice of the algorithm, by using two distinct algorithms based on different principles. The two algorithms are described in detail in Text S1 and their source code is available on line. Briefly, the Geometric Saccade Detector (GSD) detects saccades from the *x-y* planar trajectory. The Angular-Velocity based Saccade Detector (AVSD) works primarily by considering the smoothed angular heading rather than the planar position. Unless otherwise noted, the statistics shown through the paper are derived using GSD, which is *a posteriori* shown to be better suited for these particular experimental conditions and equipment. Alternative figures showing the same statistics obtained from the AVSD algorithm are available as part of Text S1.

### Capturing behavior determinism and randomness using rate-variant Poisson processes

**Figure 2:**
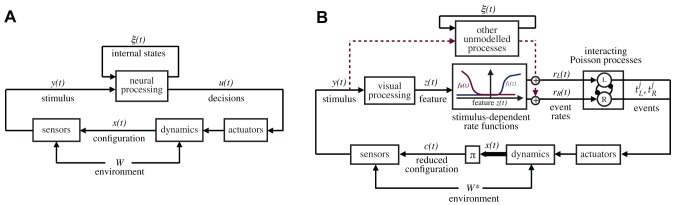
General reference models for the animal behavior and decision making. Panel A illustrates the nomenclature that we use in this paper: 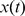 is the animal configuration (position/velocity), which ultimately depends on the past history of the animal decisions, the body dynamics, and environmental effects, here abstractly represented by the variable 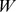. The stimulus 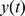 perceived by the animal is a function of the animal configuration 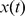 and the geometry/textures of the environment. In the most general terms, the actions of the animals, 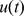, are generated on the basis of the instantaneous stimulus 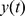 as well as the internal state 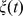, which includes, for our purposes, everything which is not observable, including metabolic and neural states. Panel B illustrates the specialization of the model that we postulate. The decisions of the animals are represented by series of observable events belonging to a fixed set of classes; in our case these are left and right body saccades. The events are assumed to be generated by a set of interacting rate-variant Poisson processes. The instantaneous rates 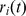 depend on several factors, including the unobservable states, and the external stimulus. The main hypothesis of this paper is that the contribution of the stimulus on the rate can be written as a function of a low-dimensional feature 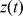 computed from the stimulus. The inference problem in this paper consists in identifying the functions 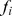 that best explain the rates as a function of the stimulus (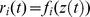). The diagram also shows the impact of other unmodeled neural processing based on internal states, acting as a disturbance in the model. We do not infer a functional description of this modeling, but we are able to bound its contribution and show that it is small with respect to the stimulus-induced contribution. The diagram also shows the *reduced configuration* 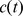, the subset of 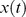 on which the stimulus actually depends. The reduced configuration depends on the particular experimental settings; in our case, we postulate that in a circular arena the stimulus is dependent on only two degrees of freedom. This is a hypothesis that can be verified a posteriori.

Figure 2A illustrates the conceptual approach of our analysis. We denote by 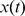 the animal's physical *spatial configuration* (its position and velocity in a fixed reference frame). The stimulus 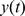 is the set of all sensory cues perceived by the animal, and it is a function of both the spatial configuration 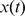 and the appearance of the world 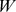. Whereas 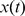 is a concrete variable that we can possibly measure, the stimulus 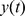 and the world 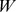 are placeholders for things that, in general, are unknown. The actions 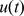 (e.g. saccades in our case) are the external manifestations of the internal neural processing, which depend both on the instantaneous stimulus as well as on 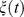, another placeholder variable that represents the animal's internal state (metabolic states, neural states, etc.), and which has dynamics of its own. We assume that it is possible to observe the spatial configuration 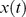 as well as infer the actions 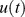 from the observations, but that the internal state 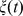 is not observable.

We make a distinction between obtaining a functional model of an animal's behavior and identifying the underlying neural processes. Obtaining a functional description of behavior means obtaining a model that can predict the actions 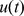 given the spatial configuration 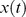 and a description of the world 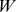. In principle, we can do this by observing an animal's behavior with enough samples of 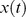, 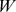 and 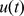. In general, however, there are a variety of neural models that could produce the same functional model. For example, many behaviors appear to be well-localized in time, suggesting an “action potential” neural model, but the underlying neural model can have very different properties [62] (in other words, the microscopic explanation might be quite different than what the macroscopic observations suggest). The model that we now describe and that we will identify should be interpreted as a purely functional model, which can inform the search for neural models, to make sure that they are compatible with the externally observable free flight behavior.

Figure 2B shows the particular model that we use in this paper. It is a particular form of the general model discussed above (Figure 2A). In this model, we propose that the animal's actions 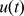 can be summarized by the saccade events. We divide the saccade events in two classes: left and right saccades. In principle, one would want to consider additional attributes of the saccades, such as speed, duration, and amplitude. The analysis might also be expanded to consider other easily identifiable events [63]. However, limiting ourselves to a binary characterization of saccades allows us to model the behavior generation as Poisson processes, which offers relatively easy inference. We model saccade generation using rate-variant Poisson processes, i.e., we assume that, for each class of events, internal and external factors influence a time-varying event rate according to a quantitative relation that we will attempt to identify.

The most important assumption of our method (which can and will be verified *a posteriori*) is that, for the purpose of generating the behavior, the high-dimensional output 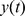 can be compressed down to a low dimensional “feature” 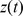. This assumption is implicit in many other previous studies, and it is informed by the knowledge of the underlying neurobiology: the first level of sensory processing in flies and other animals consists in taking a very high-dimensional sensory stream and computing the few behaviorally-relevant features from it. Our only assumption is that this low-dimensional feature exists - we do not assume that we know this feature. However, we can attempt to automatically identify this feature from the observable data. It is important to note that we do not assume to know how this feature is computed from the stimulus. Indeed, the advantage of our method is that it allows identifying this feature based only on the observable behavior, without postulating anything on the sensory processing.

Figure 2B also shows explicitly that, in addition to the feature-dependent pathway in our model, other unmodeled processing influences the behavior. The effect of this unmodeled processing will be quantitatively estimated as well. The saccade events are assumed to be generated by a set of interacting Poisson process with variable rate 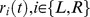. The index 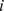 stands for either one of the two classes of events (L: left, R: right). The variable rate 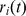 is assumed to depend both on the stimulus 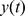 and the internal state 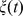, thus incorporating both random and deterministic effects. We write 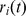 as the sum of three factors:
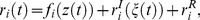
(1)where the term 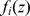 is the contribution of the external stimulus through the feature 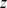; the term 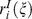 is the contribution of the internal state 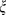; and the term 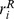 represents the contribution of a purely random stochastic process that does not depend either on an internal state or the stimulus. By omitting some of the terms in the equation above, one can recover many other simpler models. For example, purely random behavior is obtained by setting 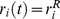.

The Poisson processes interact by inhibition. If any process generates an event, then any event generated from that process or any other process for a period of length Δ is ignored. This is meant to model a feature of many fixed action patterns that, once initiated, must run to completion before a different motor program can be initiated.

Finally, Figure 2B shows another variable 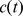, which we call “reduced configuration”. We define 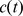 as the subset of the spatial configuration variables that actually influence the stimulus, for a particular class of environments 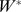. In general, for a freely flying animal, 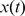 is at least a 12 dimensional quantity, including the 6 degrees of freedom for position/orientation and the corresponding 6 for velocities (additional degrees of freedom in the animal spatial configuration would be derived from the positions of body joints, such as the neck and wing positions). For particular environments, however, the stimulus is only dependent on a subset of 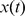. For example, if the environment is distant enough, then the visual stimulus does not depend on the forward velocity. Therefore, even though the spatial configuration 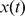 is at least 12-dimensional, actually the stimulus depends on a smaller variable 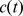, i.e., the reduced configuration.

We will show that it is possible to identify all unknowns in this model. In particular, we will identify how the feature 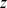 depends on the reduced configuration 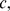 and how the rates depend on the feature. Remarkably, it is possible to do this without assumptions on how the feature 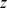 is computed from the stimulus 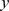 or how the stimulus 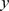 depends on the reduced configuration 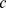. We only assume to be able to observe the reduced configuration 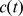 and the generated saccade events. Before describing the method, we first discuss how this model based on rate-variant Poisson processes allows us to represent different functional models.

### Predictions of different functional models

**Figure 3:**
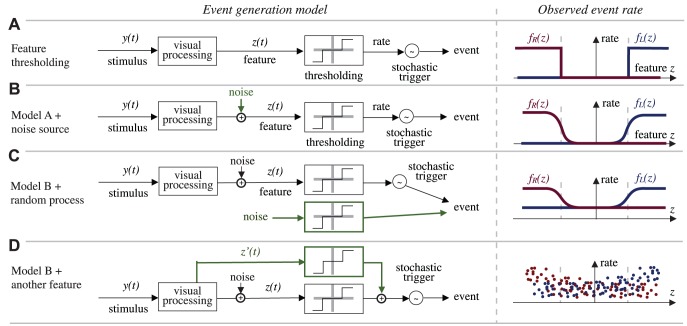
Simple models of decision making processes and relative experimental predictions. This figure shows, on the left, several simplified saccade generation schemes, and their prediction in terms of the observed statistics. All models assume that the visual stimulus 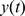 is processed as to extract a one-dimensional feature 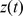 on which the animal decisions are based. The models presented are meant to represent a sample of qualitatively different functional models of behavior generations, and not necessarily biologically plausible models of neural computation. Panel A-i shows a “hard threshold” model: if the feature 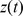 is below a threshold, no event is generated, otherwise, the event is generated stochastically with a certain rate. Panel A-ii shows what would be the prediction of the model if we were to plot the saccade generation rate (an observable quantity) as a function of the feature 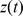, assuming we knew how to compute 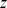. Panel B shows the same model, but with noise affecting the computation of the feature. The effect on the observed rate would be to transform the hard threshold in a soft threshold. Panel C shows a model in which there is a parallel saccade generation mechanism, which generates saccades randomly independently of the stimulus. The effect of this on the measured rate is to raise uniformly the curves. Also the contribution of some internal processing based on internal neural states which were not a function of the instantantaneous stimulus would have the same effect on the rate statistics. Panel D shows the case where the behavior depends also on some other feature of the stimulus 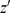 in addition to 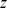. In this case, if we plotted the rates as a function of 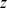, ignoring the dependency on 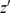, we would see that it is not possible for 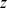 to explain the rates by itself. Therefore, once we have identified the curves 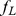, 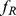, and the feature 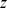, we are able to identify the contribution of a random generation process (or based on an internal state) as a uniform baseline saccade rate; and we can infer whether another feature is necessary to explain the behavior by the vertical spread of the rates.

In **Figure 3** we illustrate the predictions of four qualitatively different functional models in terms of the observed statistics. On the left side we show the functional model, and on the right we show the expected observed event rates 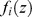 as a function of the feature 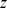. This exercise assumes that we know how to estimate the feature, which we will show later. Here we describe what we would expect to find, before embarking on the actual computation of 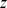.

**Figure 3**A shows a “hard threshold” model, based on the computation of a single feature 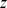, which is then thresholded to obtain the event rate. A Poisson process then generates the events based on this time variant rate. The “stochastic trigger” in the figure masks the fact that there are two processes generating two classes of events, and that these processes are interacting (see discussion above), which is not relevant to the present discussion. If the absolute value of the feature is below a threshold, no event is generated; otherwise, saccades to the left and right are generated at a fixed rate. A large fixed rate would mean that the model is practically deterministic, with a large stimulus feature 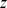 resulting in a behavioral event with only rare failures. On the right side of the figure, we show the observed event rates as a function of the feature 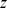. For this simple model, the observed rates as a function of the feature are straight steps. We remark that our analysis does not assume necessarily that the feature exhibits a hard threshold as in this simple model. We choose this shape merely because it allows visualizing the effect of different sources of noise.

In particular, we are interested in understanding the implications of a noise source that acts on the computation of the feature (sensory noise) compared to noise that generates behavior in a parallel process independent of the stimulus-computed feature (decision-making or motor noise).

Figure 3B shows the effect of measurement noise on the hard threshold model. Random fluctuations in the feature turn the hard threshold into a *soft* threshold.

Figure 3C shows the effect of adding a spontaneous generation process in parallel to the feature pathway. This has the effect of raising the predicted event rate by a constant value, as the parallel process is independent of the feature. A parallel generation process that depended on an unobservable internal state would have the same expected statistics if the internal state is uncorrelated with the feature. This means that a constant baseline event rate that is independent of the feature must be interpreted as the joint contribution of a purely stochastic spontaneous event generation together with a deterministic response based on internal states.

It is also important to consider the effect of another unmodeled feature 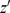 on the event rate statistics, if we only model the dependence of one feature 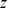. This stems primarily from practical concerns, because the dimensionality of the feature that it is possible to identify depends primarily on the amount of data available. Therefore, once the dimensionality of the feature is fixed, we need a way to judge whether that dimension is sufficient to describe the behavior.

Figure 3D augments the model of **Figure 3**A with an additional pathway that uses a different feature 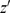. In such a case, if we plot the rates versus the feature, we will not find a clear functional dependency, indicating that the feature 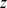 is no longer sufficient to explain the event rates. Conversely, if we find a clear functional dependency, then we can say that the feature 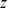 is sufficient to capture the influence of the sensory stimulus on the behavior. This does not imply that 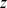 is the only behaviorally relevant feature of the stimulus, because there could be other features that are relevant for other behaviors not considered in the analysis.

Our identification algorithm, described in the next section, recovers the best one-dimensional feature 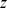 that explains the event rates. This permits constructing a function in which the experimental event rate is plotted against the feature curve. However, we anticipate that the experimental results, being dependent on experimental data, will have error bars both for dependent and independent variables. Strictly speaking, even if one finds a one-dimensional feature that uncovers a deterministic dependency between feature and rates compatible with the error bars, it is not possible to conclude that there is only one feature, because the effect of a second feature might be masked by the measurement noise. In this sense, our claims that one feature is sufficient is an application of parsimony.

In summary, we can identify the contributions of several qualitative factors by plotting the event generation rates as a function of 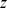. Measurement noise will soften the curve (e.g., a hard threshold is turned into a soft threshold). A parallel purely stochastic event generation process has the same effect of a deterministic process based on an internal state uncorrelated with the feature, namely it raises the curve by a fixed baseline rate independent of 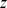. If another unmodeled feature 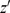 influences the behavior, there is not a strict functional dependence between the rates and the feature 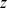.

### Identification of the feature 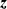


We devised a procedure that obtains an estimate of the best one-dimensional feature of the input that predicts the observed event rates. We explain here the basic idea, and provide details in Text S1. Intuitively, the feature and event rates can be obtained from the spatial statistics of the observed behavioral output. With respect to the discussion so far, the main conceptual step consists in translating the problem from the time to the space domain. So far, we have written the feature 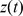 as a time-varying quantity. We have also assumed that 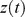 depends on the stimulus 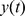, and that the stimulus depends on the animal spatial configuration 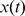, or more precisely, on the reduced configuration 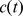. Therefore, we rewrite our model writing 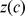 instead of 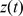. The quantity 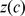 is a spatial field that we interpret as the feature computed from the typical stimulus experienced at the reduced configuration 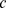. We will fit a model of the kind:
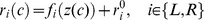
(2)where 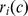 is the average event rate for the 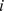-th class (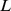: left, *R*: right) observed at the reduced configuration 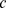; 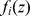 denotes the event generation rates for left and right saccades as a function of the feature, and 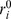 is constant term that we call *baseline event rate*.

Note the differences with respect to the previous model (Eq. 1). First, we have written the rates as a function of the reduced spatial configuration instead of time. Moreover, we do not model explicitly the contribution of the internal state. As argued above, given that we cannot measure the unobservable internal states 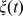, we cannot distinguish between a purely stochastic contribution and the contribution of an internal state

Therefore, the constant term 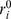 will be an estimate of the joint contribution of the two terms that we cannot distinguish:
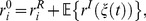
(3)where 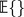 indicates the expected value taken over the whole trajectory.

We summarize here the three main phases for estimating 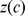 from the behavioral data, while leaving the details to Text S1. First, the reduced configuration space 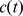 is discretized into spatial cells with a resolution that depends on the amount of data available. For each of these cells, basic statistics are computed, such as the average time spent in each cell, as well as the observed event rates in the cell. One advantage of the algorithm is that these spatial statistics, averaged over the whole trajectory, are intrinsically robust to measurement noise and uncertainty in the event detection algorithm. Next, the event *generation* rates 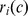 are computed from the *observed* rates. Because we assume that the Poisson processes interact with each other, and therefore the statistics of each process cannot be processed separately, and appropriate steps are required to take into account the interaction.

Once the average event generation rates 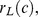 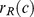 are estimated, then we find the feature field 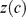 that explains both event generation rates, in the sense that there exist two functions 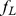 and 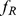 such that the constraint described by equation (2) holds. Writing the constraint explicitly for each cell 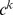, and letting 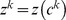 the value of the feature to estimate, we can see that we have a system of constraints of the kind:
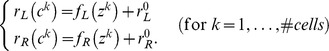
The generated event rates 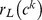 and 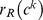 on the left side have already been estimated, while both the feature value 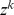 and 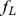 and 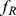 have to be estimated. The constants 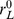 and 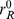 can be incorporated as part of 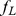 and 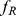. Note that this can be interpreted as a dimensionality reduction problem, because we have to find one cause (the feature 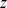) that explains two effects (left and right event rates) at the same time.

In our case, we solve a relatively simple instance of the problem in which 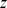 is assumed to be a scalar function. Therefore, the constraints can be algebraically manipulated to obtain a closed form solution, which also takes into account the uncertainty in all the data and provide error bars for the estimated feature. The details are given in Text S1. Our approach is very generic, and can be extended to scenarios with more than 2 behaviors and more than 1 feature.

The feature 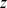 should be considered a dimensionless quantity of arbitrary scale. In fact, the equations that define it have multiple solutions. For example, suppose that 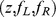 is one solution of the system of equations given by (Eq. 2). If 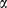 is any invertible function, then one can verify that 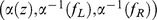 is a solution as well. Therefore, once we have obtained a solution for 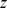, we can rescale it using any function 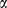 that we find convenient. In the following, we choose the rescaling function such that 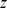 is uniformly distributed in the interval 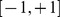.

## Results

### Event statistics and estimated feature

We tracked 88 flies for a total of 5130 seconds or approximately 1.4 hours. Of the total recorded time, we considered only the 4814 seconds of data in which the flight speed exceeded 5 centimeters per second. This threshold on the linear velocity allowed working on tracks for which saccades were easier to detect. We detected a total of 6613 saccades with this criterion, giving an average saccade rate of 1.37 saccades per second.

We chose a reduced configuration 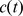 that is two-dimensional. This follows from considering only planar motion (which reduces the effective degrees of freedom to 3), and using the symmetry of the circular arena (which reduces the degrees of freedom to 2). An implied assumption (which can be verified a posteriori) is that the fly's response is not dependent on the variables not considered in the analysis; for example, even though it is known that flies [64] and other insects [65] use gaze to stabilize vision, there is no gaze variable in our model. This is because the resolution of our measurements is not enough to observe directly the relative pose of head and body, in terms of pitch, roll, or yaw. All components of the spatial configuration that are theoretically relevant for the stimulus, but cannot be measured, are “hidden” states whose contribution is lumped into the constant term in (2).

The two-dimensional reduced configuration can be parameterized in different ways, the results being independent of the particular parameterization. The primary parameterization that we use for computation uses 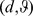 for coordinates: 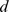 is the distance to the wall and 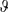 is the angle that the fly heading forms with respect to the axis of the arena (Figure 1C*i*). We chose this parameterization because it corresponds to two behaviorally relevant variables. We preferred the axis angle 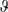 over other potentially valid representations for the heading (e.g., approach angle) because the representation is not singular, as 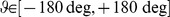 for any value of 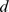.

We compute all statistics in the 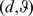 space, but we also use another choice of coordinates to visualize the same data. We rotate the original 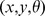 configuration of the fly around the center of the arena, such that the new coordinates are 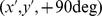. These “fly-centric” coordinates are displayed using a top-down view of the arena, in which the fly always points up (Figure 1C*ii*).

The reduced configuration 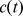 was discretized in a grid with sides of 36 cells (for 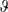) and 20 cells (for *d*) (Figure 1D*i*). The angle 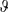 was discretized in 36 cells of equal size 10 deg. The distance 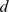 was discretized in 20 unequal intervals (note the unequal 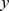 axis in Figure 1D*i*). Intervals for 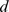 are smaller at the center of the arena and larger near the border, in such a way that each annulus of radius 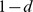 and width 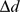 had the same area. To compensate for the sparseness of the data, each cell extends 50% into the neighbor's area. Although these choices were somewhat arbitrary, we obtain qualitatively similar results if we vary the number of the cells.

Figure 1D shows the distribution of time spent at each point of the arena, and Figure 1E shows the distribution of the detected saccades using the GSD algorithm (see Text S1 for figures using the saccades detected by the alternative AVSD algorithm). As clearly evident in Figure 1E*ii*, most of the detected saccades correspond to the fly avoiding the walls on the left or on the right. However, those are the configurations where the flies spent more time (Figure 1D). Therefore, we need to normalize this data to see the behavioral patterns.

**Figure 4:**
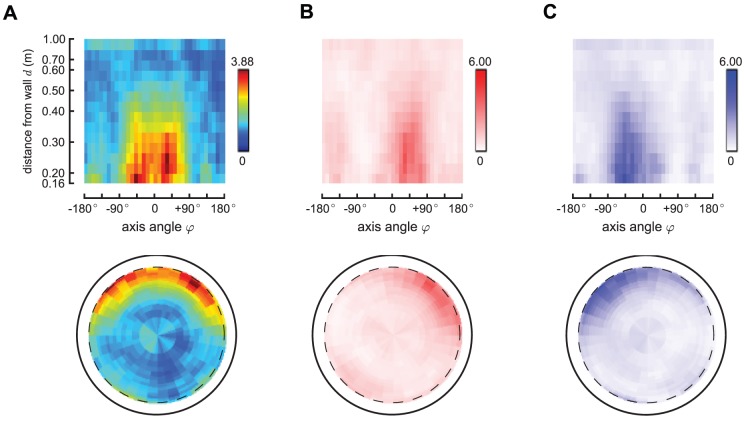
Estimated saccade generation rates. This picture shows basic statistics of the processed data. Panel A shows the estimated saccade *rate* in polar coordinates (i) and fly-centered spatial coordinates (ii), indicated in the text as 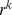. This density is obtained by taking the raw number of saccades in each cell 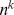
(Figure 1, Panel E), normalizing by the time spent in each cell 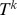 (Figure 1, Panel D), and then compensating for the interacting nature of the Poisson processes. Panels B–C show the rates for left and right saccades (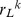 and 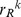, respectively), which we plot in red in Panel B (left saccades) and in blue in panel C (right saccades). Note that the left and right saccade ratios appear roughly symmetric.

**Figure 5:**
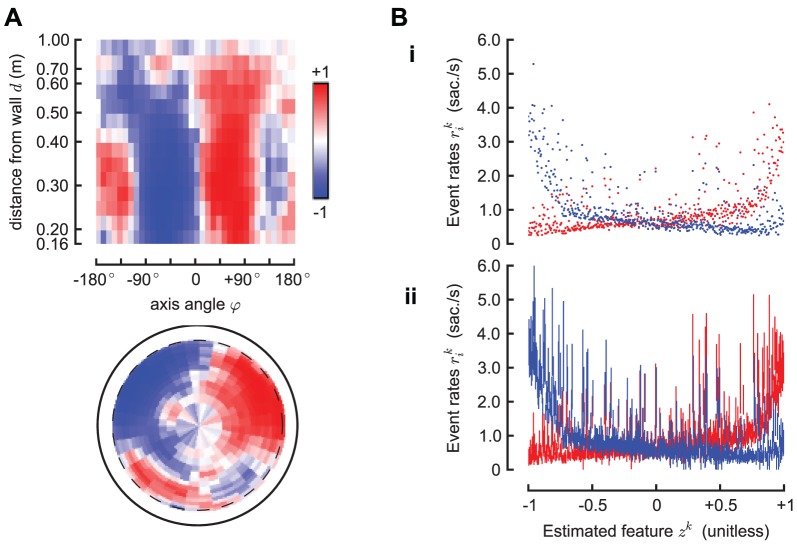
The estimated decision feature 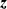
 **.** Panel A shows the estimated one dimensional feature 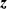. This is the best one dimensional spatial feature that explains the left and right saccade rates. It is a dimensionless quantity, which we normalize in the interval 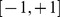. Panel B-i shows, for each cell 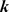, the rates 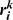 as a function of the estimated feature 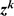; Panel B-ii shows the same data, but with error bars corresponding to 95% confidence intervals (the bars are not symmetric because the posterior distribution of the estimated rates is not Gaussian; see Supplemental Materials for details). The single feature 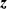 is sufficient to predict the rate in 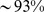 of the environment, in the sense that 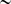
93% of the rates can be considered (with the error bars) as lying on the same curve; these curves are the functions 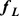 and 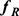 discussed previously that allow predicting the rates from the feature. The remaining 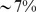 of data that this model cannot fit correspond to configurations with the fly pointing directly against the wall at a small distance (<0.3 m).

Figure 4A shows the estimated saccade generation function 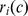 across the reduced configuration space. These rates are obtained by first computing the observed generation rates 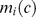 by averaging the number of saccades (Figure 1E) by the time spent in each cell (Figure 1D). Then the rates 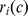 are obtained from 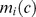 by correcting for an estimated inhibition interval 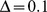 s. Panels B and C show the data separately for left and right saccades (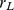 and 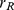). The most evident phenomenon is that the fly tends to turn left when the wall is on the right (and vice versa), however, there are many saccades of the opposite direction initiated, even when the turning would orient the fly towards the wall rather than away from it. This is the phenomenon that we want the feature 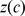 to explain: we want to find the best spatial scalar value 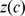 such that both 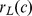 and 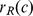 can be written as a function of 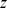. Figure 5Ai-ii shows the estimated feature 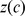 as a function of the reduced configuration c. This is the unidimensional feature that best explains both the left and saccade rates. The estimated feature using the alternative saccade detector is qualitatively similar (Figure S1). We now have the spatial feature 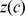 as well as the rates 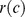 as a function of the reduced configuration 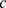 and can now plot 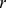 as a function of 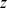 (using 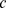 as an implicit variable). This is shown in Figure 5B, which shows, for each cell 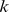, the value of 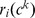 as a function of 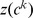. Figure 5Bi shows the data as a scatter plot, while Figure 5Bii shows the error bars on the estimated rates 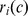 at the 95% significance level.

### Predictive power of the estimated feature

The data in Figure 5B indicate the predictive power of the feature. If the feature was perfectly predictive of the event rates, then 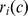 would be a function of 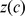. In this case, taking into account the error bounds on the rates, it is possible to find two functions 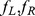 that predict the event rates in approximately 93% of the environment. More specifically, given a generic cell corresponding to the spatial configuration 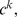 we find that the predicted event rates 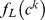 and 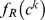 are compatible with the observed rates 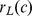 and 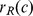 at the 95% level of significance. In practice, this means that the data in Figure 5B can be explained by two smooth curves (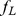 and 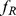) that intersect 93% of the confidence intervals corresponding to each spatial configuration. In the remaining cells (51 of 720 cells), the rates cannot be predicted by this feature alone. Further inspection (data not shown) reveals that such points correspond to configurations with the fly pointing directly against the wall at a small distance (<0.3 m). Note that being able to predict the rates from the feature does not mean that one is able to predict the direction of each single saccade event. For example, in the middle of the arena the probability of left and right saccade is 50%, and this percentage is perfectly predicted by the feature; however, it is impossible to predict the direction of the single saccade better than chance.

### Bounds on the contributions of random process and internal states

As explained before, using only external observations of the animal spatial configuration, we cannot distinguish among the contribution of a purely random endogenous saccade generation process, a deterministic process based on an internal state, any unmodeled features computed from the stimulus, and any unobservable spatial configuration that we cannot observe due to the limited resolution of our instruments. These contributions are lumped together in a baseline saccade rate. By examining the curves in Figure 5Bi we can estimate a baseline event rate 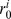 of about 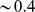 saccades/sec. By comparing with a maximum estimated event rate of 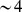 saccades/sec, we can estimate that roughly 90% of the saccades are stimulus-driven in the regions of maximum stimulus. This value depends on the geometry and texture of this particular arena (e.g., it would be different if the arena was larger or smaller). However, we predict that the baseline rate of 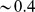
saccades/sec that we measure at the center of the arena should be independent of the geometry, as the size and textures of this arena were chosen such that the fly cannot perceive significant visual contrast from the center.

We can make some informed guesses for the contribution of the various possible processes by considering circumstantial evidence from other experiments. In tethered flight experiments, deliberately performed in the absence of salient visual stimuli, spontaneous saccade rates are on the order of 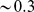 saccades/sec [66]. If we assume that these values obtained in tethered experiments are a good approximation of an assumed spontaneous generation process in free-flight, then we can account for approximately 75% of the unexplained 0.4 saccades/sec as the joint contribution of a random process and unobservable internal states. This leaves roughly 25% of unexplained data, which could possibly be explained by estimating an additional feature 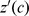, perhaps dependent on components of the spatial configuration that we cannot observe, such as the gaze direction. The contribution of a hypothetical feature 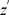 is therefore very small with respect to the contribution of the estimated 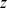, as 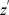 could possibly explain about 0.1 saccades/sec versus the 4 saccades/sec explained by 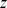.

We conclude that the saccade behavior of *Drosophila* that depends on external visual stimulus appears to depend for the most part on only a one-dimensional feature of the stimulus. These conclusions must be limited to the particular experimental condition, as we cannot exclude that more complex environments would elicit more complex responses that require a higher dimensional feature to be explained. However, even in our relatively simple flight environment, our analysis implies that the vast majority of saccades we observed are stimulus-driven and are not due to an internal, stimulus-independent search algorithm (e.g. Levy flights), as has been suggested for *Drosophila* and many other species [6]–[18].

### Approximating the feature field using known parametric structures for visual processing

We have been able to compute the feature field 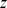 from the observable fly trajectory, without any assumptions on the fly visual processing. Nevertheless, it is interesting to test whether this independently identified feature is compatible with existing models of the first stages of visual processing in flies. In particular, we test the hypothesis of whether the identified feature can be expressed as a linear function of the perceived optic flow.

We assume the following generative model for 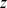:
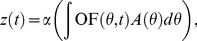
(4)where 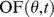 is the optic flow, or angular velocity, at the retinal angle 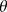 at time 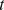, and 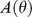 is a retinal input kernel. The value 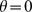 corresponds to the animal's center front visual field.

The function 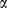 is an arbitrary nonlinear function that we include in the model, because the identification procedure allows us to know 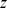 only up to a monotone transformation (i.e., if 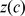 is a solution of the constraints system, then also 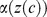 is a valid solution). We can characterize the optimal 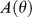 as the solution of an optimization problem:
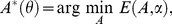
(5)where the error function 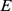 is given by:
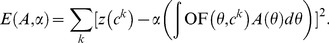
(6)In this last expression, 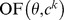 is the typical optic flow that the animal experiences at the reduced configuration 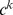. By solving this optimization problem, we try to best approximate the estimated feature over the whole environment, assuming it can be expressed as a linear function of the optic flow.

Unfortunately, we found that this optimization problem is ill posed given our data. In particular, 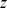 is known only at a discrete set of values 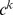 (720 cells — the density of these is constrained by the finite amount of data that we have), and it is quite noisy, whereas the unknowns 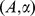 are of high dimension. Given that the resolution of the fly's visual system is around 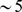
deg, it makes sense to use at least 70 numbers (∼330/5) for representing 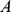. Furthermore, 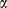 can be any monotonic nonlinear function.

**Figure 6:**
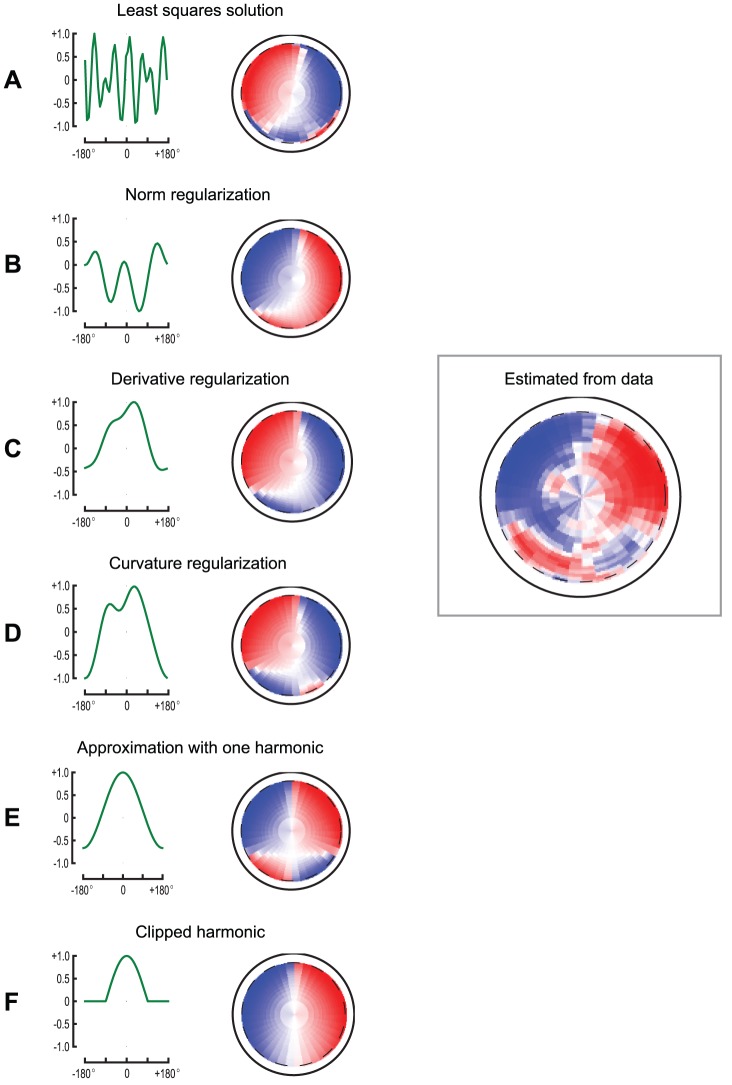
Receptive fields of wide-field motion detection consistent with the feature 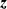
 **.** These pictures show several receptive fields of wide field motion sensitive cells, the spatial feature that they compute, as well as a comparison with the feature 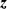 identified from the data. The pictures in first column show the kernel 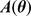; the pictures in the second column show the corresponding feature field. The panels A through D show the kernels obtained as solutions of an optimization problem, respectively by solving a linear least-squares problem (panel A), and three different regularization problems: by penalizing the norm of the solution (panel B), by penalizing the norm of the spatial derivative (panel C), and by penalizing the curvature of the solution (panel D). Note all solutions are asymmetric due to the noise in the data. Panel E shows the kernel 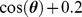, which is the closest harmonic function to the regularized solution in panel D. Panel F shows the result obtained by setting to zero this kernel in the back of the field of view. This shows that the contribution of the back of the field of view is necessary to recreate the small sidelobes of the estimated feature field.

We tried to improve the results by penalizing large values and large spatial variations of 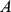 (measured either by the spatial derivatives 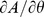 or 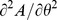). The modified error function is:
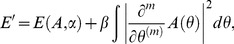
(7)for 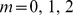 and different values of 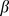. In general, by varying 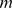 and 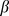, we found a multitude of solutions, all very different from each other, having approximately the same predictive power (Figure 6). We noticed that for increasing regularization values the estimated linear kernel tended to be shaped as an harmonic function, as illustrated by the kernel obtained by regularizing the second derivative (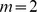) and using a large value of 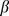 (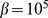), shown in Figure 6D. This kernel is still asymmetric. If we impose that the kernel must be symmetric, we find that the best approximation using one harmonic is:
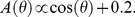
(8)This kernel and relative feature field is shown in Figure 6E, and it is a good approximation of the feature estimated from the data.

We conclude that the identified feature can be expressed as a simple function of the optic flow. However, while obtaining the behaviorally relevant feature 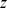 from the external observations alone is a well-posed mathematical problem, finding the function that maps the stimulus 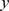 to the feature 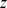 is an ill-posed problem, because the set of possible models is of very high dimension compared with the data that we have. Note that these issues are already evident when considering only linear functions of the optic flow, and would be even more pressing if we were to add other nonlinear components to the model that are known to exist in the neural circuits of the fly.

Most of the estimated kernels obtained using some form of regularization share a particular feature: 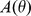 is never 0 for 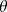 corresponding to the back of the animal, but has opposite sign in the front of the animal (Figure 6B,C,D,E). Further investigation shows that these non-zero values in the caudal region are responsible for the two small side lobes that appear in the feature field when plotted in fly-centric coordinates. If the kernel is set to zero in the back, these side lobes disappear. This is apparent by comparing the feature field in Figure 6F (corresponding to the kernel 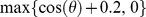) with that in Figure 6E. These results suggest that the optic flow in the back of the animal influences the fly's turning decisions. This response cannot be interpreted as pure obstacle avoidance, given that flies tend to fly forward and obstacles in the back are not expected to represent a threat for collision. For convex environments, the saccades initiated from this response would tend to align the fly's course in parallel to the environment boundaries and the overall result is to *follow* walls rather than completely avoid them (similar behavior has been observed in bees [67]). Such a visuo-motor system might provide a functional advantage with respect to the balance of collision avoidance and object search. An animal that balances attraction and obstacle avoidance would tend to remain relatively close to interesting visual features, whereas an animal whose primary reflex is to fly away from visual features would tend to find itself in large open areas, far from potential landmarks or food sources. The only way of quantitatively verifying this *attraction-deflection* hypothesis would be to obtain data from experiments within larger environments with more varied visual features. These results are also compatible with the observation in previous experiments on tethered flies that the optomotor response can be written as the function of a kernel in which the rear and front visual fields give opposite contributions [68], suggesting that a similar visual feature might be used for both behaviors.

## Discussion

In this paper, we introduced a novel method to obtain an estimate of a low-dimensional feature of the stimulus that best predicts the observable behavioral event generation rates. The feature can be obtained from observable quantities, such as the recording of the trajectory of the animal, without any assumption on the nature of the stimulus and its underlying neural processing. Using this method, we have concluded that most of the saccade events generated by fruit flies exploring a structured laboratory environment are induced by visual stimuli, and that the instantaneous stimulus can be compressed down to a one-dimensional feature, while still being predictive of the event rates in 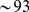
% of the environment. Using this method, it is not possible to distinguish between the contribution of an endogenous random process and a deterministic contribution dependent on an unobservable state. However, we can bound the contributions of these two terms in a baseline saccade rate that we estimate at 0.4 saccades/sec, roughly a tenth of the maximum rate. The strength of this method is that the feature 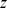 can be estimated working backwards from an animal's actions, rather than forward by postulating a model for the stimulus 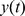 and guessing what is the relevant feature. Once we know 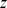, as a second step, it is possible to attempt to fit a parametric representation of neural processing to find the forward function from 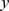 to 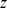, based on other assumptions about sensory processing, though this is not guaranteed to be a well posed problem, as one must optimize over all plausible models compatible with the animal's biology. In this particular case, we have shown that the feature 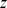 responsible for turning decisions in *Drosophila* can be written as a linear function of the optic flow, and that the particular linear kernel we obtain is compatible with that identified in tethered conditions, for a particular choice of regularization penalty to make the problem well posed. Conversely, finding 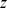 from the behavioral data is a well posed and intuitive problem, because it can be understood as a dimensionality reduction problem (find the one feature that explains multiple behaviors).

The main advantage of this approach, compared with previous methods, is that it can be applied to freely moving animals, and thus permits asking about responses to naturally important stimuli. Moreover, it does not need any assumption of linearity between some aspect of the stimulus and response, a precondition strongly needed in techniques such as reverse correlation [48]. Even advanced reverse correlation techniques in single sensory neurons [69] are not easy to generalize into models of network functionality that could be used to predict behavior.

In the future, this method could be applied to different behaviors of the fruit fly and other animals [70]. The formalization is quite generic, though some generalizations are possible. The algorithm documented in Text S1 assumes that the feature is one-dimensional in order to obtain a closed-form solution. To identify a feature of higher dimension, this must be generalized, for example by using one of the various more computationally expensive dimensionality reduction algorithms in machine learning (e.g., [71]). In any case, the rate-variant interacting Poisson process model seems apt for modeling many other behaviors (e.g., landing, taking off) that can be reliably localized in time (i.e., they have a clear beginning and end), and that can be caused by both external and internal causes.

Thinking in terms of the feature 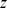 as a proxy of the stimulus can potentially be useful in understanding how different sensory modalities contribute to the same behavior. The feature is independent of the sensory modality because it is just a function of the animal configuration, and it is a proxy of the typical stimulus perceived at the location, so it could be used to study, for example, the influence of olfaction instead of visual processing on turning behavior, or their interaction, which has been the object of much research [72]–[75] [56]. Note, however, that we do have the strong assumption that the stimulus is a constant function of the configuration, so the framework cannot be easily extended to time-varying stimuli.

This approach might also be useful to study different behaviors at the same time. *Drosophila* has a large repertoire of behaviors/reflexes which are stimulus-triggered, such as landing, take-off, chasing mates, and escaping from small targets. In this case, we focused on saccades, and we found the feature 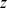 encoding the relevant function of the stimulus for saccade decisions. If one repeated the analysis for a different behavior (e.g., landing), there would likely be another feature 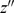, that would be different from 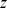. However, if this was repeated for all fly behaviors, one would find that at some point the new identified features would be redundant; for example, in the case of vision, the number of features is upper bounded by the number of upstream signals towards the lobula. Ultimately, this exercise might provide a prediction of whether two behaviors are likely to share the same neural pathways.

Potentially, this technique could help in quantifying the behavioral differences of different genotypes. This model makes a distinction between the feature 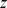 and the event generation rate functions 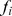. Whereas z is assumed to be correlated with computed percepts, 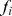 might be correlated more with the motor functions. This distinction could be used to obtain insight regarding the function of genetic manipulations such as a screen in which populations of neurons are “silenced” with a hyperpolarizing ion channel or synaptic release blockade. For example, if a modified animal gives the same feature 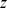 but modified rate functions 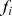, it would be evidence that the silenced neurons are involved with motor generation rather than with stimulus processing. Consequently, with a large-scale screen [76], [77], it might be possible to obtain a classification of phenotypes into sensory, decision making, and motor deficits. Similarly, we could use this feature to quantitatively compare the properties of different species.

Another interesting but more substantial extension of this work would be to expand the mathematical formalism to incorporate measurements of neuronal activity into the internal processing structure. This is now done in freely moving worms [78], [79], [32] and zebrafish [80]; in adult flies, most neural recording during behavior is being done on fixed flies [81]–[83], [42].

## Supporting Information

Figure S1
**The estimated decision feature** 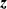 **(results obtained with alternative detection algorithm).** This figure shows the equivalent results of Figure 5 using the AVSD algorithm for saccade detection instead of GSD. Compared with Figure 5, the graphs are similar in many respects. Panel A shows the estimated one dimensional feature 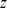 as a function of the reduced configuration. Note that there are only two areas, corresponding to positive and negative 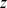, instead of four, as in Figure 5A. Panel B shows the observed event rates as a function of the estimated feature. Compared to Figure 5B, in this case the estimated feature 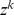 is slightly less predictive of the rates 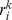. This means that the events detected by the AVSD algorithm cannot be correlated with the stimulus as well as those detected by AVSD. This suggests that, for this particular data, the GSD algorithm is able to better detect behaviorally relevant events in the trajectory data.(EPS)

Text S1 **Mathematical details of the identification technique and implementation details of the saccade detection algorithms.**
(PDF)
