# Supplementary material for: Discriminating External and Internal Causes for Heading Changes in Freely Flying Drosophila
Source: PLoS Comput Biol. 2013 Feb 28;9(2):e1002891. doi: 10.1371/journal.pcbi.1002891 (PMC3585425; doi:10.1371/journal.pcbi.1002891)
Supplement: Text S1 — Mathematical details of the identification technique and implementation details of the saccade detection algorithms. (PDF) [file pcbi.1002891.s002.pdf]

DISCRIMINATING EXTERNAL AND INTERNAL CAUSES FOR HEADING CHANGES IN FREELY FLYING *DROSOPHILA*

ANDREA CENSI, ANDREW D. STRAW, ROSALYN W. SAYAMAN, RICHARD M. MURRAY, MICHAEL H. DICKINSON

Text S1

Contents

1. Problem formalization 2
2. Modeling assumptions 5
3. Observability analysis 7
4. Estimation of event generation rates 8
5. Feature identification 10
6. Algorithm summary 16
7. Details of saccade detection algorithms 17
8. Guide to the experimental results 18
9. Complete plots (Geometric saccade detector) 22
10. Complete plots (Angular-velocity based detector) 28
References 33

1. Problem formalization

There are 5 kinds of objects in our analysis: *states*, *stimuli*, *features*, *events*, and *models*.

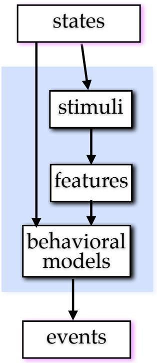

FIGURE 1.1.

states States can be both *observable* and *unobservable.* This distinction is largely practical in nature, and dependent on the technology available at the moment.

Examples of observable states: position/velocity of the animal.

Examples of unobservable states: metabolic states, neural states.

stimuli These are all the sensory cues available to the animal for decision making. Stimuli are a function of the states. In this work, stimuli are abstract entities: they are assumed to exist, as a function of the states, but they are never computationally manipulated.

Examples of stimuli: perceived luminance at each photoreceptor, perceived odor traces, perceived acceleration.

features We define "features" as the behaviorally-relevant low-dimensional functions of the stimuli that are used for decision making. The existence of these features is postulated. The theoretical justification to investigate low-dimensional functions of the stimuli is that, while the stimuli are very high-dimensional, the decisions are usually very low-dimensional. Therefore, only a low-dimensional feature of the stimuli can possibly contribute to behaviors.

Example of feature: left/right optic flow imbalance.

(behavioral) events Behavioral events (or simply: events) are the external manifestations of decision making that we can observe. Our formulation applies to behaviors that can be clearly identified in time.

Examples of events: start of a saccades, landing, taking off.

(behavioral) models These are generative models that explain the observed events, as a function of the external stimuli and the internal states.

1.1. Behavioral pathway of interest in this work.

Naturally, a complete understanding of animal behavior can only be attained by considering all possible stimuli and all observable behaviors. However, the complexity of a model that can be reliably identified is bounded by the amount, diversity, and quality of the data that can be collected. Therefore, in practice, one can only consider a limited "behavioral pathway" that interests only a subset of the stimuli and a subset of the behaviors. We limit our analysis to the visual stimulus, which is a function of the animal position, and we take discrete body saccade events as the observable manifestation of behavior.

Whether the particular stimulus considered (visual stimulus) is sufficient to build a model for the chosen behavior is something that is not justified *a priori*, but rather will have to be confirmed *a posteriori* by the analysis.

1.2. Observable states.

We call $x(t) \in X$ the observable animal *configuration*, which includes the position and linear/angular velocity in 3D space, and X the *configuration space*. The space X has dimension 12 (6 degrees of freedom for position/orientation, plus corresponding velocities).

An estimate of $x(t)$ is provided by our tracking software. While the observations are occasionally very noisy, we assume that $x(t)$ is observable, and we do not model the noise on x in this analysis. In the following, it will be clear that this does not impact the analysis much, as the method is very robust to bounded noise on $x(t)$.

The tracking system provides data at 60Hz. We ignore this time discretization and assume for convenience that $t \in \mathbb{R}$.

TABLE 1. Symbols used in this paper

Symbol	*meaning*
$t \in \mathbb{R}$	Time index.
$x(t) \in X$	Observable configuration (position and velocity).
$\xi(t)$	Unobservable states.
$y(t)$	Perceived stimuli.
C	Reduced configuration space
$c(t) \in C$	Reduced configuration at time t.
$r_i(t)$	Instantaneous event generation rates.
Δ	Inhibition interval.
$i \in \{L, R\}$	Index over event classes.
$t_i^1, t_i^2, \dots, t_i^j, \dots$	Detected events of the i-th class ($i \in \{L, R\}$).
j	Index over discrete events in time
$b_i(t)$	Detected events as a series of impulses in time.
f_i	Event generation rate function.
$\delta(t - \bar{t})$	Impulse function centered at $\bar{t}$.
$\{C^k\}_{k=1}^K$	Partition of C in K cells.
$k \in [1, K]$	Index over cells.
$c^k \in C$	Center of k-th cell.
n_i^k	Number of detected events in k-th cell.
n^k	Total number of detected events in k-th cell.
m_i^k	Measured event rate in the k-th cell.
r_i^k	Average event generation rate for i-th behavior in k-th cell.
$\boldsymbol{r}_i = \{r_i^k\}_{k=1}^K$	Vector containing all event generation rates over cells.
z^k	Feature associated to k-th cell.
$\boldsymbol{z} = \{z^k\}_{k=1}^K$	Vector containing all feature values over cells.
order	Function computing the order of each element in a vector.
$a \circ b$	Composition of the functions a and b.
$\mathsf{Diff}(\mathbb{R})$	Diffeomorphisms of $\mathbb{R}$; set of strictly monotone functions.
$\mathcal{N}(\mu, \sigma)$	Gaussian distribution with mean μ and standard deviation σ.
$\mathsf{Unif}(a, b)$	Uniform distribution on the interval $[a, b]$.

1.3. **Unobservable states.** We call $\xi(t)$ the set of all other states that are behaviorally relevant but not observable:

$$\xi(t) \triangleq \text{all behaviorally relevant states} \quad \setminus \quad x(t).$$

These unobservable states include:

- unobservable kinematic properties (e.g., angle of fly neck);
- metabolic states that influence behavior (e.g., hunger);
- other unmodeled properties of the environment (residual odor traces, etc.).

1.4. **Behavioral events.** The observable behavioral events that we consider are the so-called "body saccades", or just "saccades". These are the moments where a fly turns rapidly in the horizontal plane.

There are two classes of events: left (L) and right (R) saccades. We limit our analysis to only the saccade direction; in principle, there are many observable properties of a saccade that would be interesting to analyze (top angular velocity, amplitude, etc.); those are left for future work.

The index "i" will be used to index over event classes: $i \in \{L, R\}$. Every time we write a formula with the generic index i, it is understood that $i \in \{L, R\}$.

The criterion for defining a saccade and the algorithms for detecting it are discussed in Section 7. Here, we assume that we have a procedure that, given a trajectory $x(t)$, returns a series of detected saccade events, represented by their initiation times $\{t_i^j\}$:

$$\text{detected left saccades:} \qquad t_L^1, t_L^2, \dots, t_L^j, \dots$$
$$\text{detected right saccades:} \qquad t_R^1, t_R^2, \dots, t_R^j, \dots$$

The index "j" is an index over the sequence of events.

We assume that these events are observable (i.e. we detect all saccades perfectly). In practice, we found that our saccade detection algorithms give false positives or false negatives on less than 1% of the data against a subset of the data manually annotated; this error can be neglected for our analysis, because all the statistics that we compute are essentially averages over spatial patches, therefore robust to random failures of the detection procedure.

We also introduce the variables $b_L(t), b_R(t)$. These variables are an alternative representation of the event sequences as trains of impulses:

$$b_i(t) = \sum_j \delta(t - t_i^j).$$

Here, $\delta(t - \bar{t})$ is the impulse centered at time $\bar{t}$. This notation will be convenient for further manipulations.

1.5. **Stimulus and reduced configuration.** We call $y(t)$ the set of all external stimuli perceived by the animal at time t. We assume that $y(t)$ is a function h of the animal configuration $x(t)$, corrupted by a noise process $\nu(t)$:

$$y(t) = h(x(t), \nu(t)).$$

In our case, $y(t)$ would be the luminance perceived on the retina, plus odor traces, and other sensory cues depending on position. The stimulus is also a function of the environment shape/textures/etc, which in the paper is indicated as "W". However, because the environment is considered fixed, we omit it from the formulation.

We do not assume that $y(t)$ is observable, nor we are interested in estimating it; rather, we use this variable only as theoretical device to formalize our intuition of *environment symmetry*. We say that an environment has a symmetry if there exist two distinct points $x_1, x_2 \in X$ such that

$$h(x_1, \nu) = h(x_2, \nu).$$

If this is the case, then it makes sense to compress the state $x(t) \in X$, to a smaller, minimal representation $c(t) \in C$. We call $c(t)$ the *reduced configuration*, and C the *reduced configuration space*. We assume that C is given, along with a projection map $\pi : X \to C$, which maps the point $x(t)$ to $\pi(x(t)) = c(t) \in C$. In other words, the map π extracts from the whole state $x(t)$ the variables that are necessary to determine the stimulus. Moreover, we assume that $c(t)$ is a minimal representation, in the sense that, for all points x_1, x_2:

$$\pi(x_1) = \pi(x_2) \qquad \Leftrightarrow \qquad h(x_1, \nu) = h(x_2, \nu).$$

In other words, $c(t)$ is sufficient to compute the stimulus (if one knew the function h), and it cannot be further reduced to an even smaller representation.

In our particular case, we have a circular symmetry, as the environment is a circular arena with uniform patterns on the walls. If one assumes X to describe position, orientation and linear/angular velocity of the animal, the circular symmetry allows to decrease by 1 the number of degrees of freedom of the data, by projecting the 12-D space X to a 11-D space. The reduced configuration stimulus depends on the details of each experiment and on the experimenter's assumptions. For example, even if the arena was circular, the symmetry would not be valid if there were non symmetric patterns on the walls.

We consider the reduced configuration space and the projection map as other modeling choices whose validity must be justified *a posteriori*. Our assumptions regarding the reduced configuration space are explained in Section 2.5.

2. Modeling assumptions

This section introduces all modeling assumptions of our analysis and the implied approximations.

2.1. **Behavioral events are generated by time-variant interacting Poisson processes.** We model the generation of observable events as a set of interacting Poisson processes with time-variant rates $r_i(t)$ and inhibition interval Δ:

$$\{b_i(t)\}_{i\in\{L,R\}} \sim \text{InteractingPoisson}(\{r_i(t)\}_{i\in\{L,R\}}, \Delta).$$

This means that, once any process generates one event at time t, no other event can be generated in the interval $[t, t+\Delta]$, from the same or another process. A Poisson process with time-variant rate is sometimes called a Cox process [1].

2.1.1. *Interpretation of the inhibition interval* Δ. The inhibition time is meant to model the fact that we consider instantaneous events that correspond to the initiation of a motor program, and one motor program must complete before another can be initiated.

2.2. **The event generation rates depend on the instantaneous stimulus, unobservable states, and endogenous random process.** We assume that event generation rate $r_i(t)$ can be written as the sum of three terms r_i^S, r_i^E, r_i^R, which model the contributions of the stimulus, the unobservable states, and an endogenous random process:

$$r_i(t) = \underbrace{r_i^S(y(t))}_{\substack{\text{instantaneous}\\ \text{stimulus}\\ \text{contribution}}} + \underbrace{r_i^E(\xi(t))}_{\substack{\text{unobservable}\\ \text{states}\\ \text{contribution}}} + \underbrace{r_i^R}_{\substack{\text{endogeneous}\\ \text{random process}\\ \text{contribution}}}. \tag{2.1}$$

2.3. **The high-dimensional stimulus y can be compressed down to a smaller feature z.** The term $r_i^S(y)$ models the contribution of the stimulus to the event generation. We assume that this contribution can be written as a function of a low-dimensional feature $z \in Z$ of the stimulus y:

$$r_i^S(y) = f_i(z(y)).$$

We call the functions f_i the *event generation rates functions*.

2.4. **The feature has monotone effect on the two behaviors.** We introduce another constraint on the two functions f_R, f_L that allows to obtain a closed-form solution to the identification problem. This assumption is very specific to the particular problem studied. We assume that:

- The function f_L is monotonically increasing:

$$f_L(z_1) > f_L(z_2) \Leftrightarrow z_1 > z_2. \tag{2.2}$$

- The function f_R is monotonically decreasing:

$$f_R(z_1) > f_R(z_2) \Leftrightarrow z_1 < z_2. \tag{2.3}$$

These assumptions can be verified a posteriori after fitting the data.

2.5. **Choice of the reduced configuration space C.** Choosing the reduced configuration space C is a critical step of the analysis. There is a clear tradeoff: if the C space is too small, then we cannot represent the variability of behavior. If it is too large, then the data that we have will not be dense enough to have accurate statistics. This is the reason we do not use directly the values of $x(t)$.

In our case, we project the original 12 degrees of freedom space X to only two. The original degrees of freedom are, position (3), attitude (3), linear velocity (3), and angular velocity (3). We make the following assumptions:

1. The altitude can be ignored (removes 1 dof for the z position, and the corresponding 1 dof for velocity).
2. As for the attitude, only the yaw is relevant (removes 2 dof).

(3) The angular velocity is ignored; the saccades start when the fly is flying approximately straight (removes 3 dof) .
(4) The fly has negligible sideway velocity (removes 1 dof).
(5) The linear forward velocity is not relevant (removes 1 dof).

These assumptions bring the degrees of freedom from 12 to 3. The circular symmetry of the arena allows to further reduce the analysis to a 2D space. There is no particular algorithmic complication if the space has more than 2 dimensions. Given the amount of data we have, a 2D space allows the recorded trajectories to fill the space densely enough to compute the statistics necessary.

Note that the particular *parametrization* of the space C does not matter. In the paper we use two different parametrizations to display the results.

2.6. **From time-variant to timeless spatial quantities.** In the following, it will be convenient to write the rates r_i^S as a function of the configuration rather than as function of time. This is possible because we have assumed that $y(t)$ is a function of the reduced configuration $c(t) \in C$.

Consequently, also the feature z is a function of the reduced configuration. We call $\gamma : C \to Z$ the function that assigns a value of the feature to each reduced configuration:

$$z = \gamma(c).$$

2.7. **Reduced model identified.** While we assume (2.1) as the true model for the rate r_i, we will identify a reduced model that lumps together the contributions of the unobservable states and the endogenous random process. We cannot distinguish the contributions of r_i^E and r_i^R because we cannot access the unobservable states. However, we can show that we can still identify the stimulus-dependent term r_i^S by averaging over the trajectories.

We will identify the model:

$$r_i(c) = \underbrace{f_i(z(c))}_{\substack{\text{instantaneous}\\ \text{feature}\\ \text{contribution}}} + \underbrace{r_0}_{\substack{\text{unobservable states}\\ \text{+endogeneous process}\\ \text{average contribution}}}, \tag{2.4}$$

where r_0 lumps together the contribution unobservable states and endogenous process.

To arrive at (2.4) from (2.1), we just need to average over time. Fix a point c_* and let C_* be a small area containing c_*. Define $\mathbb{E}_{c(t)\in C_*}\{\cdots\}$ as the average of a quantity limited to those times in which $c(t) \in C_*$. Then, by averaging the observed instantaneous rate in C_*, we obtain

$$\mathbb{E}_{c(t)\in C_*}\{r_i(t)\} \quad = \quad \mathbb{E}_{c(t)\in C_*}\{f_i(z(c))\} + \mathbb{E}_{c(t)\in C_*}\{r_i^E(\xi(t))\} + r_i^R.$$

Define $r_i(c_*) = \mathbb{E}_{c(t)\in C_*}\{r_i(t)\}$ to be the average rates around the point c_*.

If we assume that the feature is a continuous function of the configuration and that the neighborhood C_* is small enough, then we can approximate:

$$\mathbb{E}_{c(t)\in C_*}\{f_i(z(c))\} \simeq f_i(z(c_*)).$$

If we assume that the unobservable states are uncorrelated with the external configuration, then the value of the expectation $\mathbb{E}_{c(t)\in C_*}\{r_i^E(\xi(t))\}$ does not depend on the configuration C_*:

$$\mathbb{E}_{c(t)\in C_*}\{r_i^E(\xi(t))\} = \mathbb{E}\{r_i^E(\xi(t))\} = \bar{r}_i^E$$

Therefore, by defining

$$r_0 = r_i^R + \bar{r}_i^E$$

we arrive at (2.4).

3. Observability analysis

Our first step towards identification of the model is an observability analysis. We first give some remarks on the dimensionality of the feature that we can identify; then we show that, fixed the dimensionality, the problem is still underconstrained because there are multiple solutions that satisfy the constraints.

3.1. **Dimensionality of the feature.** We can show that the dimension of the feature z such that the problem is well posed is bounded by the dimension of the reduced configuration space C and the number of event classes.

Proposition 1. *For the identification problem to be well-posed, we must have*

$$\dim(Z) < \min\{\dim(C), n_{\text{events}}\}.$$

In particular, for two event classes and a two-dimensional configuration space, we can only estimate a one-dimensional feature.

Proof. Our sets of constraints is

$$\begin{cases} z & = \gamma(c), \\ r_i^S(c) & = f_i(\gamma(c)), \qquad i = 1, \dots, n_{\text{events}}. \end{cases}$$

Geometrically, we have that the function γ maps C to Z, and then the function $\boldsymbol{f} = \{f_i\}$ maps Z to $\mathbb{R}^{n_{\text{events}}}$:

$$C \xrightarrow{\gamma} Z \xrightarrow{f} \mathbb{R}^{n_{\text{events}}}.$$

For the estimation problem to be well posed, we need to have the hourglass structure

$$\dim(C) > \dim(Z) < \dim(\mathbb{R}^{n_{\text{events}}}),$$

otherwise there are some trivial solutions.

(1) In the case
$$\dim(C) = \dim(Z) < \dim(\mathbb{R}^{n_{\text{events}}}),$$
we can choose $\gamma =$ Identity and $f_i(z) = r_i^S(c)$ as a trivial solution.

(2) In the case
$$\dim(C) > \dim(Z) = \dim(\mathbb{R}^{n_{\text{events}}}),$$
we can choose $f_i =$ Identity, $z(c) = \{r_i^S(c)\}$ as a trivial solution.

□

3.2. **Observability.** The model is not fully observable, in the sense that we can find multiple solutions for the parameters that fit the data equally well. This is formalized in the following proposition.

Proposition 2. *Suppose that the configuration space is discretized in K cells, each with center $c^k \in C$. Let the bold vectors $\boldsymbol{r}_L, \boldsymbol{r}_R \in \mathbb{R}_+^K$ represent event generation rates associated to different animal configurations ($\boldsymbol{r}_i = \{r_i^k\}$, where k ranges over configuration), and similarly let $\boldsymbol{z} = \{z^k\}$ be the feature associated to the configurations $\boldsymbol{c} = \{c^k\}$. Assume that the model postulated so far holds exactly. Then we can write the constraints in vector form as:*

$$\boldsymbol{r}_L = f_L(\boldsymbol{z}), \tag{3.1}$$

$$\boldsymbol{r}_R = f_R(\boldsymbol{z}),$$

$$\boldsymbol{z} = \gamma(\boldsymbol{c}). \tag{3.2}$$

Assume that the rates $\boldsymbol{r}_L$, $\boldsymbol{r}_R$ and the reduced configurations $\boldsymbol{c}$ are observable, and that the functions f_L, f_R, γ are unknown a priori. Then it is possible to estimate $\boldsymbol{z}$ only up to a monotone transformation, in the sense that it is not possible to distinguish between a solution $\boldsymbol{z}_1$ and a solution $\boldsymbol{z}_2$, if $\boldsymbol{z}_1 = \alpha(\boldsymbol{z}_2)$ where $\alpha : \mathbb{R} \to \mathbb{R}$ is a strictly monotone function. Consequently, one cannot distinguish between the event generation rate functions (f_L, f_R) and $(f_L \circ \alpha^{-1}, f_R \circ \alpha^{-1})$, where "$\circ$" denotes function compositions.

Proof. A "solution" of the system is a tuple $(f_L, f_R, \gamma, \boldsymbol{z})$ for which the constraints (3.1)–(3.2) hold. If the system of constraints was completely observable, there could be only one solution. However, suppose $s = (f_L, f_R, \gamma, \boldsymbol{z})$ is a solution, and consider an invertible function α, and the solution tuple s^α defined as

$$\begin{aligned} s^\alpha &= (f_L^\alpha, f_R^\alpha, \gamma^\alpha, \boldsymbol{z}^\alpha) \\ &= (f_L \circ \alpha^{-1}, f_R \circ \alpha^{-1}, \alpha \circ \gamma, \alpha(\boldsymbol{z})). \end{aligned}$$

One can verify that, assuming s is a solution, s^α is another solution of the system, because it respects the constraints (3.1)–(3.2):

$$\begin{aligned} r_L &= f_L(\boldsymbol{z}) = f_L(\alpha^{-1}(\alpha(\boldsymbol{z}))) = f_L^\alpha(z^\alpha), \\ r_R &= f_R(\boldsymbol{z}) = f_R(\alpha^{-1}(\alpha(\boldsymbol{z}))) = f_R^\alpha(z^\alpha), \\ z^\alpha &= \alpha(z) = \alpha(\gamma(c)) = \gamma^\alpha(\boldsymbol{c}). \end{aligned}$$

□

This is the formal way to show that there is an ambiguity. A more intuitive way to see the same thing is by rewriting (3.1)–(3.2) in a slightly different way. Knowing γ is equivalent to knowing z, so we can write the constraints in terms of γ only:

$$\begin{aligned} \boldsymbol{r}_L &= f_L(\gamma(\boldsymbol{c})), \\ \boldsymbol{r}_R &= f_R(\gamma(\boldsymbol{c})). \end{aligned} \tag{3.3}$$

Intuitively, we have "2 equations for 3 variables"; because γ always appears composed together with f_L and f_R, it cannot be observed independently.

3.3. **Interpretation of the unobservability.** If the reduced feature is defined only up to a monotone transformation, then it should not be thought as a physical quantity, or as a measure of physiological activity. In fact, we cannot associate a meaningful measurement unit to it. Rather, the feature represents an *ordering* of the configurations (in fact, the order is what is conserved by any monotone function). All we can say is whether in a certain configuration the feature is weaker or stronger than in another.

Still, from the point of view of the analysis and the visualization, it is useful to consider z as a real-valued quantity associated to each configuration. From the point of view of the estimation, we cannot distinguish between $\boldsymbol{z}$ and $\alpha(\boldsymbol{z})$; therefore, we can choose any particular function α for visualizing the feature. We will choose a function α so that $\boldsymbol{z}$ varies between -1 and $+1$ across the environment.

4. Estimation of event generation rates

In the first part of the algorithm, we estimate the event generation rates $\{r_i^k\}$, where $i \in \{L, R\}$, and k ranges over configurations. This operation is slightly more complicated than just dividing the number of detected events by time, because the events are generated by Poisson processes that interact with each other. For example, this means that the rate of observed left saccade events depends not only on r_L, but also the rate r_R: if r_R is very high, then we expect to see fewer left saccade events, as there is more inhibition.

4.1. **Statistics collected from the data.** Divide the reduced configuration space C in cells $\{C^1, C^2, \dots, C^k, \dots C^K\}$, possibly overlapping, and each with center $c^k \in C$. It is assumed that the discretization is small enough to capture the variability of behavior, but large enough so that the samples are dense enough.

For each cell, we compute the following statistics from the data:

n_i^k The number of events of the i-th class detected in the k-th cell.
T^k The time spent in the k-th cell.

If one defines the variable $I^k(t)$ as

$$I^k(t) = \begin{cases} 1 & \text{if } c(t) \in C^k, \\ 0 & \text{otherwise.} \end{cases}, \qquad \text{for } k \in [1, K].$$

then the two quantities n_i^k and T^k can be written as follows:

$$\begin{aligned} n_i^k &= \int I^k(t) b_i(t) dt, \\ T^k &= \int I^k(t) dt. \end{aligned}$$

We define also the following auxiliary statistics:

n^k The total number of events detected in the cell:

$$n^k = \sum_i n_i^k = n_L^k + n_R^k$$

m_i^k The *measured event rates*, given by

$$m_i^k = \frac{n_i^k}{T^k}.$$

m^k The total event rate m^k per cell:

$$m^k = \sum_i m_i^k = m_L^k + m_R^k.$$

4.2. **Robustness of statistics to measurements noise.** These are all the statistics that we need from the data. Note that the position $x(t)$ need to be only accurate enough so that it can be assigned to the correct cell. In particular, we do not need to compute higher derivatives of $x(t)$, therefore operations like smoothing are not necessary (some smoothing might be necessary to detect the saccade events). Also, misdetection of saccade events does not impact much the analysis; a 1% false positive/negative rate only changes the statistics m_i^k by 1%.

4.3. **Measured saccade rates vs. saccade generation rates.** Due to the inhibition period $\Delta > 0$, the measured event rates underestimate the event generation rates: $m_i^k < r_i^k$. This is true even in the case of 1 process, and the effect is more evident with multiple processes.

4.3.1. *Event generation rate estimation for one process without inhibition.* Consider first the case of only one Poisson process $b(t)$, with event generation rate r, and no inhibition period. Suppose we observe the process for T seconds, counting n events, obtaining the measured rate $m = n/T$. Then m is the maximum likelihood estimate of r.

4.3.2. *Event generation rate estimation for one process with inhibition.* However, if there is a non-zero inhibition period, Δ, then the measured rate m underestimates the true rate. For example, the observed rate m cannot be higher than $1/\Delta$, no matter how high the rate r is, because due to inhibition we can observe at most one event every Δ seconds.

A better estimate of the rate r can be found as follows:

$$\hat{r} = \frac{n}{T - \Delta n}.$$

The interpretation is easy: if there were n events, then the process was inhibited for Δn seconds. Therefore, $T' = T - n\Delta$ is the effective time in which the process was active and could generate events.

The same formula can be written in terms of the measured rate:

$$\hat{r} = \frac{n}{T(1 - \frac{\Delta n}{T})} = \frac{m}{1 - \Delta m}.$$

Note that $r \to m$ as $\Delta \to 0$: if there is no inhibition, the measured rate is the generation rate.

4.3.3. *Event generation rate estimation for multiple processes with inhibition.* Next, consider the case in which there are multiple processes $\{b_i(t)\}$ inhibiting each other. It is easy to see that not taking into account the inhibition can strongly skew the estimate. In fact, imagine that there is one process with very high rate. The rates of the other processes will be severely underestimated because the frequent process will often inhibit them. Fortunately, also in this case we can easily normalize the rates.

Suppose we observe a series of processes $\{b_i(t)\}$ over an interval T and we count n_i events for each process. Let $n = \sum_i n_i$ be the total number of events observed. Then we know that the effective time in which the processes were not inhibited is $T - n\Delta$ (note that according to this model, it does not matter which process inhibits which). Accordingly, the event generation rates can be estimated as

$$\hat{r}_i = \frac{n_i}{T - \Delta \sum_i n_i}, \tag{4.1}$$

or, writing it as a function of the measured event rates:

$$\hat{r}_i = \frac{m_i}{1 - \Delta \sum_i m_i}. \tag{4.2}$$

Note that, as $\Delta \to 0$, $r_i \to m_i$ and the estimate of the rate of one process does not depend on the measured rates of the others. Also note that the correction factor necessary for one process depends on the cumulative intensity of all the others.

4.4. **Estimation of confidence bounds for the event generation rates.** The formulas (4.1)-(4.2) give the maximum likelihood estimators for the event generation rates. One can estimate confidence intervals using the method discussed in Guerriero *et al.* ed[2]. Upper and lower 95% confidence bounds are found as follows:

$$r \in [\underline{r}, \overline{r}] = \left[\frac{\left(1 - \frac{1.96}{\sqrt{n-1}}\right) n}{T - \Delta \sum_i n_i}, \frac{\left(1 + \frac{1.96}{\sqrt{n-1}}\right) n}{T - \Delta \sum_i n_i} \right]. \tag{4.3}$$

5. Feature identification

At this point, we assume to have an estimate of the event generation rates r_i^k. We write again our model with explicit dependence on the cell c^k:

$$\begin{aligned} r_L^k &= f_L(z^k), \\ r_R^k &= f_R(z^k), \\ z^k &= \gamma(c^k). \end{aligned}$$

Our goal is to find z. Once z is known, then the three functions f_L, f_R, γ can be recovered from these equations. For simplicity, we first assume that we know the event generation rates r_i^k precisely. Sections 5.1 and 5.4 show how to estimate z under this simplifying assumption. Then, Sections 5.3 and 5.4 show how to take into account the uncertainty in r_i^k.

5.1. **The $\mathsf{order}(\cdot)$ function and its properties.** We introduce the $\mathsf{order}(\cdot)$ function, sometimes also called "rank".

Definition 3. Define the function $\mathsf{order} : \mathbb{R}^K \to \mathsf{Perm}(K)$, which takes a vector in $\mathbb{R}^K$ and associates to it a permutation of length K, that gives the order of each element in the sequence.

For example, we would have

$$\mathsf{order}([10, 20, 30]) = [0, 1, 2],$$

and

$$\mathsf{order}([100, 3.14, 42]) = [2, 0, 1].$$

We will need three simple properties of this function.

Proposition 4. *Properties of the* order$(\cdot)$ *function:*

(1) *The order of the elements of a vector does not change if a strictly increasing function is applied to the vector.*
Let $\boldsymbol{a}, \boldsymbol{b} \in \mathbb{R}^K$*, let* $\beta : \mathbb{R} \to \mathbb{R}$ *be a strictly increasing function, and let* $b^k = \beta(a^k)$*. Then*
$$\mathsf{order}(\boldsymbol{b}) = \mathsf{order}(\beta(\boldsymbol{a})) = \mathsf{order}(\boldsymbol{a}).$$

(2) *Applying a strictly decreasing function inverts the order in the vector.*
Let $\boldsymbol{a}, \boldsymbol{w} \in \mathbb{R}^K$*, let* $\chi : \mathbb{R} \to \mathbb{R}$ *be a strictly decreasing function, and let* $w^k = \chi(a^k)$*. Then*
$$\mathsf{order}(\boldsymbol{w}) = \mathsf{order}(\chi(\boldsymbol{a})) = K - \mathsf{order}(\boldsymbol{a}).$$

(3) *Two vectors with elements in the same order are equivalent up to a increasing function.*
Let $\boldsymbol{x}, \boldsymbol{y} \in \mathbb{R}^K$ *and suppose that* $\mathsf{order}(\boldsymbol{x}) = \mathsf{order}(\boldsymbol{y})$*. Then there exists a strictly increasing function* $\psi : \mathbb{R} \to \mathbb{R}$ *such that* $y^k = \psi(x^k)$*.*

5.2. **Estimating the reduced stimulus, assuming that there are no uncertainties.** As a first propaedeutic step, assume that the values of r_K^i are known exactly without uncertainty. From the relation $\boldsymbol{r}_L = f_L(\boldsymbol{z})$, the assumption that f_L is strictly increasing, and property (1) of Proposition 4, one obtains that

$$\mathsf{order}(\boldsymbol{r}_L) = \mathsf{order}(\boldsymbol{z}). \tag{5.1}$$

By applying the order function on both sides of the equality, we were able to simplify f_L from the expressions, because applying a strictly increasing function does not change the order of the data.

Similarly, from the relation $\boldsymbol{r}_R = f_R(\boldsymbol{z})$, the assumption that f_R is strictly decreasing, and property (2) in Proposition 4, one obtains that

$$\mathsf{order}(\boldsymbol{r}_R) = K - \mathsf{order}(\boldsymbol{z}). \tag{5.2}$$

Here, because f_R is decreasing, the order of the elements is reversed.

At this point, we can use (5.1) and (5.2) to obtain an overdetermined system of equations for $\mathsf{order}(\boldsymbol{z})$. The least square solution is obtained by simply averaging the two terms:

$$\begin{aligned} \widehat{\mathsf{order}}(\boldsymbol{z}) &= \text{estimate of } \mathsf{order}(\boldsymbol{z}) \\ &= \frac{1}{2}\mathsf{order}(\boldsymbol{r}_L) + \frac{1}{2}(K - \mathsf{order}(\boldsymbol{r}_R)) \\ &= \frac{1}{2}\mathsf{order}(\boldsymbol{r}_L) + \frac{1}{2}\mathsf{order}(-\boldsymbol{r}_R). \end{aligned} \tag{5.3}$$

By property (3) of Proposition (4), we know that knowing $\mathsf{order}(\boldsymbol{z})$ is equivalent to knowing $\boldsymbol{z}$ up to a diffeomorphism:

$$\mathsf{order}(\boldsymbol{z}) = \gamma(\boldsymbol{z}) \qquad \text{for some } \gamma \in \mathsf{Diff}(\mathbb{R}).$$

By the observability analysis of the problem (Proposition 2), we know that we can estimate $\boldsymbol{z}$ only up to a diffeomorphism, therefore we are done and use as our estimate of the feature:

$$\hat{\boldsymbol{z}} = \widehat{\mathsf{order}}(\boldsymbol{z}).$$

Because $\hat{\boldsymbol{z}}$ is determined only up to a diffeomorphism, for purely esthetic reasons we can normalize it in the $[-1, +1]$ range, by setting

$$\hat{\boldsymbol{z}}' = \frac{\widehat{\mathsf{order}}(\boldsymbol{z}) - K/2}{K}. \tag{5.4}$$

Once we know an estimate of $\boldsymbol{z}$, the shape of the functions f_L and f_R can be obtained directly from the relations $\boldsymbol{r}_L = f_L(\boldsymbol{z})$, $\boldsymbol{r}_R = f_R(\boldsymbol{z})$.

This simplified procedure is valid only if the values $\boldsymbol{r}_i$ are known without uncertainty. If uncertainty is present, then a slightly more complicated computation is needed, described in the next section. Most of the difficulty arises in understanding how uncertainty propagates through the order function.

5.3. **Estimating the ranks of a collection of random variables.** We now put uncertainty back in the picture. The values $\boldsymbol{r}_L, \boldsymbol{r}_R$ are not known precisely; rather, we only have a posterior distribution estimated from the data. Here we want to show that the order function is very sensitive to noise, therefore the approximation (5.3) cannot be used directly.

We want to solve the following problem: given a collection of K random variables $\boldsymbol{X} = \{X^k\}_{k=1}^K$ with known probability distribution , estimate $\mathsf{order}(\boldsymbol{X})$.

There are several unsatisfactory ways to solve this problem. One could just use the mean of the distributions to estimate the relative order:

$$\mathsf{order}(\boldsymbol{X}) = \mathsf{order}(\mathbb{E}\{\boldsymbol{X}\}). \tag{5.5}$$

However, this estimate is not satisfactory because it does not take into account the variance of the variables. An example of this situation is shown in the panels of Fig. 5.1. In this figure, we simulate a collection of random variables $\boldsymbol{X} = \{X^k\}$, where each random variable has a uniform distribution over an interval of length 0.1 with center $\exp(-0.02\,k)$:

$$X^k \sim \text{Uniform}(\exp(-0.02\,k) - 0.05, \exp(-0.02\,k) + 0.05)$$

The probability distribution of the variables is represented in Fig. 5.1a. The result of computing (5.5) is shown in Fig. 5.1b. The plot is a straight line because the means of the variables are already ordered by k.

Fig. 5.1c shows two realizations $\boldsymbol{x}_1, \boldsymbol{x}_2$ of the random variables $\boldsymbol{X}$, while Fig. 5.1d shows the order of the realizations $\mathsf{order}(\boldsymbol{x}_1)$ and $\mathsf{order}(\boldsymbol{x}_2)$. As one can see, the order of the variables changes dramatically, especially for large k, where the means of consecutive variables are very similar. This shows that applying the order operation to $\boldsymbol{r}$ as in (5.3) is not a sensible way to estimate $\mathsf{order}(\boldsymbol{r})$ if the data is noisy.

A reasonable estimate of $\mathsf{order}(\boldsymbol{X})$, along with error bounds, can be obtained by simulation. Suppose that the distribution of $\boldsymbol{X}$ is known:

$$p(X^k = x) = \Theta^k(x),$$

with Θ^k a known probability distribution. Then one can compute the distribution of $\mathsf{order}(\boldsymbol{X})$ simply by drawing samples of $\boldsymbol{X}$ from the known distribution and computing the observed order. More in detail, one computes a set of n samples $\boldsymbol{x}_1, \boldsymbol{x}_2, \dots, \boldsymbol{x}_n$ all with the same distribution: $\boldsymbol{x}_i = \text{sample}(\boldsymbol{\Theta})$. Then the distribution of $\mathsf{order}(\boldsymbol{X})$ can be approximated by the samples $\{\mathsf{order}(x_i)\}$. In particular, we can derive mean and confidence bounds. This method is summarized as Algorithm 1.

Remark 5. This method is very simple and requires only the ability to draw samples from the distribution of $\boldsymbol{X}$. For completeness, we briefly mention the analytical difficulties to

Algorithm 1 OrderBySampling

Input:

$\boldsymbol{\Theta} = \{\Theta^k\}_{k=1}^K$ The probability distributions of K random variables $\boldsymbol{X} = \{X^k\}_{k=1}^K$, such that $X^k \sim \Theta^k$.

Parameters:

N Number of simulations.

Output:

$\Psi = \{\Psi^k\}_{k=1}^K$: An estimate of the probability distribution of $\mathsf{order}(\boldsymbol{X})$.

Algorithm:

function $\Psi = \mathsf{OrderBySampling}(\boldsymbol{\Theta}, N)$:

(1) For j in $1, \dots, N$:
   (a) Draw the sample $\boldsymbol{x}_j \sim \boldsymbol{\Theta}$.
   (b) Compute $\boldsymbol{o}_j = \mathsf{order}(\boldsymbol{x}_j)$
(2) Compute the density Ψ^k as the observed distribution of $\{o_j^k\}_{k=1}^N$.

obtaining a more analytical solution. (Skipping this remark does not impact understanding of the rest of this document). The reader will have noticed that we did not give an analytical characterization of the distribution of $\mathsf{order}(\boldsymbol{X})$. In theory, the distribution of $\mathsf{order}(\boldsymbol{X})$ can be thought as a sum of binomial distributions. In fact, we have:

$$\begin{aligned} \mathsf{order}(X^k) &= \text{number of variables in } \{X^j\}_{j=1}^K \text{such that } X^k \geq X^j \\ &= \sum_{j=1}^K b_{jk}, \end{aligned}$$

where b_{jk} is a binomial variable defined as

$$b_{jk} = \begin{cases} 1 & \text{if } X^j \geq X^k, \\ 0 & \text{if } X^j < X^k. \end{cases}$$

The problem is that the variables b_{jk} are *not* independent. Therefore all the convenient theoretical results about sums of independent binomials cannot be used. Assuming that the distribution of $\boldsymbol{X}$ is uni-modal, one can expect the distribution of $\mathsf{order}(\boldsymbol{X})$ to be uni-modal as well, and if the number of variables K is large enough, a Gaussian approximation could be appropriate. For example, Fig. 5.1 shows the distribution of $\mathsf{order}(X^{10})$, $\mathsf{order}(X^{15})$, and $\mathsf{order}(X^{40})$.

5.4. **Estimating the reduced stimulus, taking into account the uncertainty in $\boldsymbol{r}$.** We now refine the procedure in Section 5.4 taking into account the uncertainty in $\boldsymbol{r}$. Let Θ_L^k, Θ_R^k be the posterior distributions of r_L^k, r_R^k estimated from the data:

$$\begin{aligned} p(r_L^k \mid b_L) &= \Theta_L^k, \\ p(r_R^k \mid b_R) &= \Theta_R^k. \end{aligned}$$

Using Algorithm 1, we can estimate the distribution of $\mathsf{order}(r_L^k), \mathsf{order}(r_R^k)$; call the resulting distributions Φ_L^k and Φ_R^k.

$$\begin{aligned} p(\mathsf{order}^k(\boldsymbol{r}_L) \mid b_L) &= \Phi_L^k = \mathsf{OrderBySampling}(\Theta_L^k), \\ p(\mathsf{order}^k(\boldsymbol{r}_R) \mid b_R) &= \Phi_R^k = \mathsf{OrderBySampling}(\Theta_R^k). \end{aligned}$$

From (5.1) and (5.2) we can compute the corresponding probability distributions of $\mathsf{order}(\boldsymbol{z})$, which we call Γ_L^k and Γ_R^k:

$$\Gamma_L^k(m) = p(\mathsf{order}^k(\boldsymbol{z}) = m \mid b_L) = \Phi_L^k(m), \tag{5.6}$$

$$\Gamma_L^k(m) = p(\mathsf{order}^k(\boldsymbol{z}) = m \mid b_R) = \Phi_R^k(K - m). \tag{5.7}$$

We use a Gaussian approximation for these densities:

$$\Gamma_L^k \simeq \mathcal{N}(\mu_L^k, \sigma_L^{2\,k}), \tag{5.8}$$

$$\Gamma_R^k \simeq \mathcal{N}(\mu_R^k, \sigma_R^{2\,k}). \tag{5.9}$$

Assuming a non-informative prior on $\mathsf{order}^k(\boldsymbol{z})$:

$$p(\mathsf{order}^k(\boldsymbol{z}) = m) = 1/K,$$

we can find the posterior distribution of $\mathsf{order}(\boldsymbol{z})$ by fusing together (5.8)-(5.9), obtaining

$$\mathsf{order}^k(\boldsymbol{z}) \sim \mathcal{N}(\mu^k, \sigma^{2\,k}), \quad \text{with} \tag{5.10}$$

$$\begin{aligned} \mu^k &= \left(\frac{1}{\sigma_L^{2\,k}} + \frac{1}{\sigma_R^{2\,k}} \right)^{-1} \left(\frac{\mu_L^k}{\sigma_L^{2\,k}} + \frac{\mu_R^k}{\sigma_R^{2\,k}} \right), \\ \sigma^{k\,2} &= \left(\frac{1}{\sigma_L^{2\,k}} + \frac{1}{\sigma_R^{2\,k}} \right)^{-1}. \end{aligned}$$

Normalize the feature distribution in the $[-1,+1]$ range:

$$\overline{\mu}^k = \frac{\mu^k - K/2}{K}. \qquad (5.11)$$

$$\overline{\sigma}^k = \frac{\mu^k}{K}.$$

This gives the best estimate for the feature z^k.

At this point we can estimate the functions f_L, f_R directly using the relations

$$r_L^k = f_L(z^k) \qquad \text{and} \qquad r_R^k = f_R(z^k).$$

Note that the shape of f_L, f_R can be visualized directly by plotting the points (z^k, r_R^k) and (z^k, r_R^k), along with confidence intervals (for both r_L^k, r_R^k and z^k), and it is not strictly necessary to impose some parametric form.

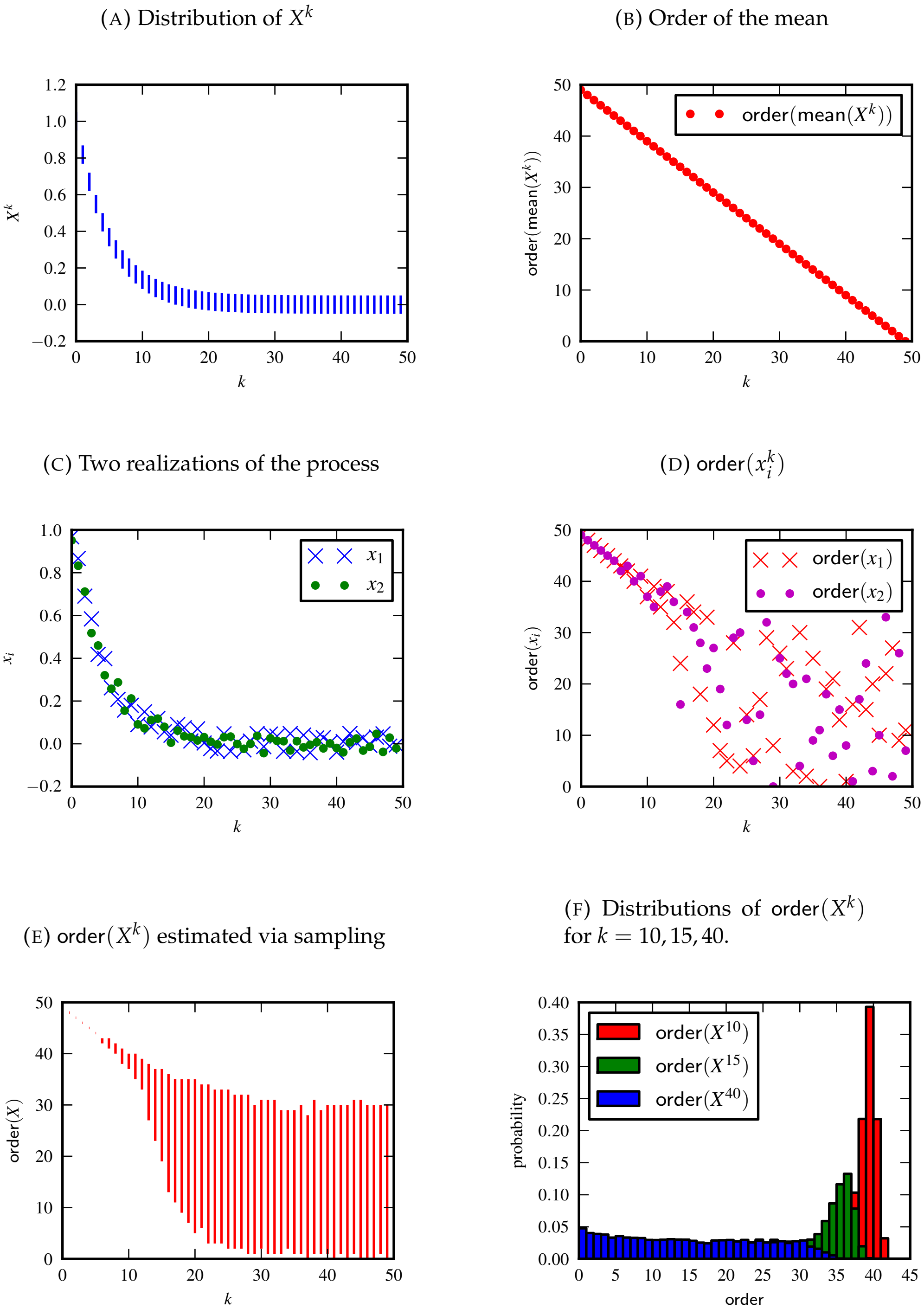

FIGURE 5.1. Synthetic data used to illustrate the properties of the order function and the order estimation procedure of Algorithm 1.

6. Algorithm summary

This is a summary of the identification algorithm.

Input:

$x(t) \in X$	Recorded configuration.
t_i^j	The series of detected events, where $i \in \{L, R\}$ ranges over behaviors and j ranges over events.

Parameters:

$\pi : X \to C$	Projection map from the configuration space to the reduced configuration space.
$\{C^k\}_{k=1}^K$	Partition of the reduced configuration space C in cells: $C = \cup_{k=1}^K C^K$.

Output:

$\{z^k\}_{k=1}^K$	Reconstructed feature over the reduced configuration space.
f_L, f_R	Reconstructed event generation rate functions.

Procedure:

(1) Compute the reduced configuration $c(t)$ using the projection π :

$$c(t) = \pi(x(t)).$$

(2) Define the variable $I^k(t)$ as 1 if the animal is in the k-th cell at time t:

$$I^k(t) = \begin{cases} 1 & \text{if } c(t) \in C^k, \\ 0 & \text{otherwise.} \end{cases}, \qquad \text{for } k \in [1, K].$$

Compute the permanence time in each cell:

$$T^k = \int I^k(t) dt, \qquad \text{for } k \in [1, \ldots, K].$$

(3) Define $b_i(t)$ as a series of impulses centered at the observed events t_i^j:

$$b_i(t) = \sum_j \delta(t - t_i^j), \qquad i \in \{L, R\}.$$

Count the number of events observed in each cell:

$$n_i^k = \int I^k(t) b_i(t) dt, \qquad \text{for } i \in \{L, R\},\ k \in [1, K].$$

(4) Compute the observed event rates m_L^k, m_R^k:

$$m_i^k = \frac{n_i^k}{T^k}, \qquad \text{for } i \in \{L, R\},\ k \in [1, K].$$

(5) Estimate the inhibition interval Δ from the inter-event statistics.

(6) Estimate the *event generation rates* using

$$r_i^k = \frac{m_i^k}{1 - \Delta \sum_i m_i^k}, \qquad \text{for } i \in \{L, R\},\ k \in [1, K].$$

Let the bold symbol $\boldsymbol{r}_i$ indicate the set of values for all cells:

$$\boldsymbol{r}_i = \{r_i^k\}_{k=1}^K.$$

(7) *Deterministic approximation:*
  (a) Compute an estimate of $\mathsf{order}(\boldsymbol{z})$ using (5.3).
  (b) Compute the normalized feature using (5.4).
  (c) Having estimated z^k and r_L^k, r_R^k, fit the functions f_L, f_R directly from the relations $r_L^k = f_L(z^k)$; and $r_R^k = f_R(z^k)$.

(8) *Method taking into account the uncertainty of the data:*
  (a) Compute 95% confidence intervals $[\underline{r}_i^k, \bar{r}_i^k]$ for r_i^k using (4.3):

$$[\underline{r}_i^k, \bar{r}_i^k] = \left[\frac{\left(1 - 1.96/\sqrt{n_i^k - 1}\right) n_i^k}{T^k - \Delta \sum_i n_i^k}, \frac{\left(1 + 1.96/\sqrt{n_i^k - 1}\right) n_i^k}{T^k - \Delta \sum_i n_i^k} \right].$$

  Take $\Theta_i^k = \mathrm{Unif}([\underline{r}_i^k, \bar{r}_i^k])$ as an approximation of $p(r_i^k | b_i)$.

(b) Estimate the probability distribution $\Phi_i^k = p(\mathsf{order}^k(\boldsymbol{r}_i)|b_i)$ using the method described as Algorithm 1:

$$\{\Phi_i^k\}_{k=1}^K = \mathsf{OrderBySampling}(\{\Theta_i^k\}_{k=1}^K).$$

(c) Compute the distributions $\Gamma_i^k = p(\mathsf{order}^k(\boldsymbol{z}))$ from Φ_i^k using (5.6)–(5.7).
(d) Approximate Γ_i^k as a Normal distribution $\mathcal{N}\left(\mu_i^k, \sigma_i^{2\,k}\right)$ using mean and variance.
(e) Compute the posterior distribution $\mathcal{N}(\mu^k, \sigma^{2\,k})$ for z^k using (5.10).
(f) Normalize the values of the feature in the $[-1, +1]$ range using (5.11) obtaining $\mathcal{N}(\overline{\mu}^k, \overline{\sigma}^k)$, which is our final estimate for z^k.
(g) Having estimated z^k and r_L^k, r_R^k (both with appropriate confidence bounds), estimate f_L, f_R directly from the relations $r_L^k = f_L(z^k)$; and $r_R^k = f_R(z^k)$.

7. Details of saccade detection algorithms

The Python source code for both algorithms is available online at
http://github.com/AndreaCensi/geometric_saccade_detector
The Kalman filter/smoother implementation are available as part of Flydra.

7.1. **Geometric saccade detector (GSD).**

The geometric saccade detector (GSD) algorithm works using x, y tracking data, rather than using angular velocity. This makes it most useful for noisy data, as it does not need to derive the data twice (once to obtain the linear velocity, and again to obtain the angular velocity). However, it cannot be used for tethered experiments, for which the x, y data is not available.

The algorithm can be summarized as follows:

(1) Obtain the trajectory $p(k) = \langle x(k), y(k) \rangle$.
We use the trajectory returned by Flydra which has been processed with a causal Kalman filter. The algorithm is robust enough to be used on noisy data; so we do not use smoothing.
(2) Consider separately each instant $\bar{k}$.
(a) Translate the coordinate frame, such that $p(\bar{k}) = \langle 0, 0 \rangle$ becomes the origin.
(b) Fix an interval Δ and consider the samples in $[\bar{k} - \Delta, \bar{k} - 1] \cup [\bar{k} + 1, \bar{k} + \Delta]$. Δ is a parameter which, for our data, is set to $\Delta = 5$ time steps ($\simeq 0.07s$).
(c) Compute the polar coordinates of the samples with respect to the origin:

$$\alpha(k) = \arctan 2(y(k), x(k))$$

(d) Compute the average orientations before and after:

$$\theta_{\text{before}}(\bar{k}) = \frac{1}{\Delta} \sum_{k=\bar{k}+1}^{\bar{k}+\Delta} \alpha(k)$$

$$\theta_{\text{after}}(\bar{k}) = \frac{1}{\Delta} \sum_{k=\bar{k}-\Delta}^{\bar{k}-1} \alpha(k)$$

In these computations, we consider that angles are defined modulo 360°.
(e) Similarly, compute the dispersion $\sigma_{\text{before}}(\bar{k})$, $\sigma_{\text{after}}(\bar{k})$.
(f) Define the amplitude of a potential saccade as

$$A(\bar{k}) = \theta_{\text{after}}(\bar{k}) - \theta_{\text{before}}(\bar{k})$$

and the "score" of a saccade as

$$S(\bar{k}) = \sigma_{\text{before}}(\bar{k}) + \sigma_{\text{after}}(\bar{k}).$$

(3) Mark potential saccades as the points where

$$S(\bar{k}) \geq S_{\min} = 20°$$

and

$$A(\bar{k}) \geq A_{\min} = 20°.$$

(4) At this point, we have a sequence $S(\bar{k})$ that describes the likelihood that there is a saccade at time $\bar{k}$. To segment the data, examine each point $\bar{k}$ in decreasing order of S, and mark the points in the interval $[\bar{k} - \Delta, \bar{k} + \Delta]$ as unavailable as well. Repeat until all points are marked unavailable.

7.2. **Angular-velocity based saccade detector (AVSD).** The AVSD algorithm operates using the angular velocity. The advantage of this algorithm is that it can be used also for tethered data, where only the animal heading is available. However, if one starts with x, y data, then one must derive the data twice to obtain the angular velocity.

The algorithm can be summarized as follows:

(1) Obtain the angular velocity $\omega(k)$.
In our case, this is done using a Kalman smoother on the position data, then deriving once to obtain the translational velocity, obtain the angular heading as the planar direction of the velocity vector, then derive again to obtain the angular velocity.
(2) Define saccades as the intervals where $|\omega(k)| > \omega_{\min}$.
In our case, set $\omega_{\min} = 300\,\mathrm{deg/s}$.

8. Guide to the experimental results

8.1. **Configuration space.** The Flydra tracking system tracks the position and velocity of flies in the cylindrical Mamarama arena. The arena has height 0.8m and radius 1m. The data is returned at a temporal resolution of 60Hz, with spatial noise on the order of 0.5cm. Much of the complexity of the saccade detection algorithm (explained in Section 7) is due to handling this limited resolution. However, for the sake of simplicity, we are going to ignore these issues in this section. We then consider the data to be a continuous signal.

We define the following quantities:

$p(t) \in \mathbb{R}^3$ Position of the animal with respect to a fixed coordinate frame.
$v(t) \in \mathbb{R}^3$ Linear velocity.
$R(t) \in SO(3)$ Attitude (represented as a rotation matrix).
$\omega(t) \in \mathbb{R}^3$ Angular velocity.

These quantities constitute the original observable configuration $x(t)$ of dimension 12:

$$x(t) = \langle p(t), v(t), R(t), \omega(t) \rangle .$$

8.2. **Reduced configuration space.** As explained in Section 2.5, we project down the data to a reduced configuration space C of dimension 2. This is done in two steps. In the first step, we only assume that the planar configuration of the animal is relevant. This reduced the configuration to $\langle p_1(t), p_2(t), \theta(t) \rangle$, where p_1 and p_2 are the planar components of the position, and θ is the planar orientation. This reduces the dimension from 12 to 3. By an arbitrary choice of reference frame, we let $p_1 = 0, p_2 = 0$ correspond to the center of the arena.

The second step consists in taking into account the symmetry of the environment. Due to the circular symmetry of the environment, we assume that the animal perceives approximately the same stimulus if its planar configuration is rotated around the center of the arena. Therefore, the two variables that contribute to the stimulus are the distance from the center and the animal orientation.

More formally, we choose as reduced configuration space the two variables d, φ defined as follows:

$$\begin{aligned} d &= 1 - \sqrt{p_1^2 + p_2^2}, \\ \varphi &= \theta - \arctan 2(p_2, p_1) \end{aligned}$$

The angle φ, which we call *axis angle*, is the angle that the animal heading forms with the axis that joins the animal position to the arena center. These are the two quantities that are invariant to a rotation around the center of the arena.

The reduced configuration is defined as $c = (d, \varphi) \in C$. The bounds of the domain C are as follows:

$$\begin{aligned} d &\in [0.15, 1\text{m}], \\ \varphi &\in [-180\text{deg}, 180\text{deg}]. \end{aligned}$$

We express angles in degrees. Note that all the operations on φ are to be executed modulo 360deg ($\varphi = 180$ and $\varphi = -180$ are the same point).

For the distance d, we have:

$$d = \begin{cases} 1 & \text{fly at the center of the arena} \\ 0.15 & \text{limit for reliable data} \\ 0 & \text{fly landed on the wall.} \end{cases}$$

We censor the data at around $d \geq 0.15$. Albeit the tracking system returns data in the whole domain of the arena, it is sometimes unreliable at $d \leq 0.15$, as sometimes the tracking cannot be obtained with full quality, notwithstanding the use of 11 cameras. In the interval $d \geq 0.15$, the data is always very reliable and of homogeneous quality.

For the axis angle, we have

$$\varphi = \begin{cases} -180° & \text{fly pointing directly away from the wall} \\ -90° & \text{closest point on the wall is at the } \textbf{left} \\ 0 & \text{fly pointing towards the closest point on the wall} \\ +90° & \text{closest point on the wall is at the } \textbf{right} \\ +180° & \text{fly pointing directly away from the wall} \end{cases}$$

Fig. 8.1a shows an example plot in these coordinate. Note that the discretization for d is not uniform in $[0, 1]$, but it is chosen such that each cell in the φ, d space corresponds to an equal area in the p_1, p_2, θ space.

8.3. **Fly-centric coordinate space.** The $c = (\varphi, d)$ space is the space where we collect statistics and do all of our computations. For visualizing the results in a more intuitive way, we use also another representation, which is just a change of coordinate, shown in Fig. 8.1b. In this representation we use two "aligned" spatial coordinates x^a, y^a. The animal always points "up".

More formally, in these coordinates the dynamics of the animal is given by:

$$\begin{aligned} \frac{d}{dt} x^a(t) &= 0, \\ \frac{d}{dt} y^a(t) &> 0. \end{aligned}$$

The change of coordinates is given by:

$$\begin{bmatrix} x^a \\ y^a \end{bmatrix} = \begin{bmatrix} \cos(-\theta) & -\sin(-\theta) \\ \sin(-\theta) & \cos(-\theta) \end{bmatrix} \begin{bmatrix} p_1 \\ p_2 \end{bmatrix}.$$

This means that the original configuration $\langle p_1, p_2, \theta \rangle$ is rotated around the arena center to obtain the configuration $\langle x^a, y^a, 0 \rangle$.

8.4. **On the choice of coordinates.** When going from the configuration (φ, d) to (x^a, y^a), there is a singularity at the center of the arena. Uniform cells in the (φ, d) domain appear as elongated "pizza slices" in the (x^a, y^a) domain. This appears as a slight artifact when the cells obtained in the (φ, d) domain are plotted in the (x^a, y^a) domain.

Note that all the operations done in the analysis are invariant to the choice of the coordinates. However, once chosen one or the other, one cannot avoid a singularity in the change of coordinates when converting between the two systems. Our rationale for choosing the

(φ, d) domain is that these variables are behaviorally relevant even for environments with different geometry (e.g., rectangular). This should make future comparisons with experiments with different environment easier.

8.5. **Nuisances in the analysis.** Finally, we summarize all the approximations/limitations of this analysis, which should be kept in mind when interpreting the results; Fig. 8.2 contains a diagrams highlighting some of the factors.

(1) There might be unobservable states that influence behaviors. The contribution of these states appears as a baseline event rate not explained by the feature.

(2) The reduced configuration space C might be too small to be a proxy for the true configuration. If this is true, then the estimated features cannot be predictive of all events.

For example, we ignore the altitude and the velocity of the fly.

(3) The dimension of the feature identified is bounded by the number of event classes considered. To identify a feature of dimension n, one needs at least $n + 1$ events. Therefore, the stimulus might contain more behaviorally-relevant information than what is revealed just by the feature identified from the particular event classes considered.

(4) There are measurements errors:

(A) Axis angle (φ) / distance from wall (d) plot.

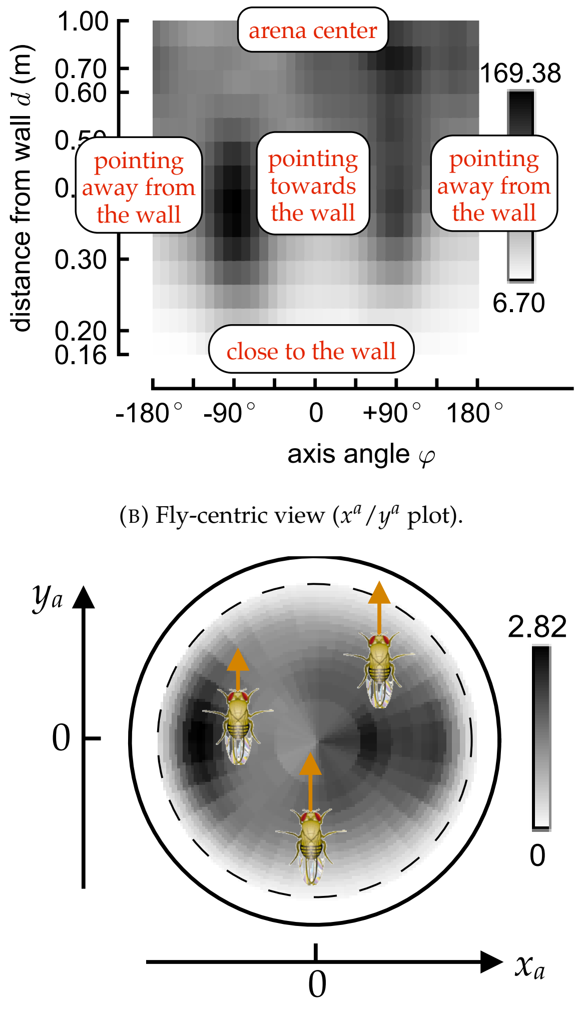

(B) Fly-centric view (x^a/y^a plot).

FIGURE 8.1. Explanation of the two kinds of plots used.

- The state $x(t)$ is noisily observed, and the noise is not negligible with respect to the partition of C.
- The events are not exactly detected.

The analysis is generally robust to this kind of noises, but still they are unmodeled phenomena.

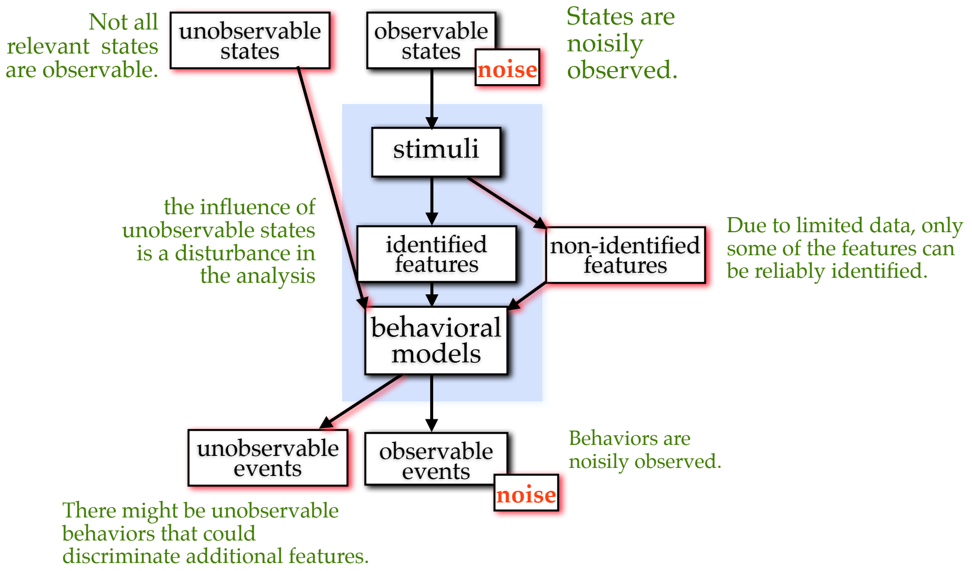

FIGURE 8.2. Nuisances in the analysis

9. Complete plots (Geometric saccade detector)

The next pages show the complete statistics using the events detected by the GSD algorithm.

FIGURE 9.1. Time spent in each cell

(A) φ, d space (B) x^a, y^a space

Time spent in cell

T^k

(s)

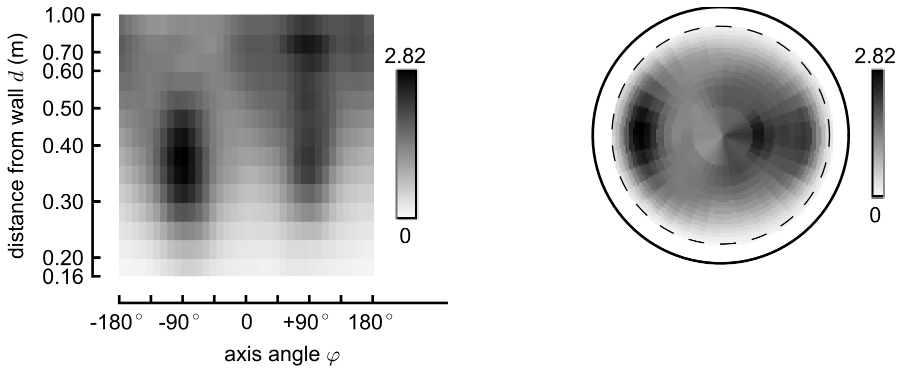

FIGURE 9.2. Mean speed (whole trajectory)

(A) φ, d space (B) x^a, y^a space

Mean speed

(m/s)

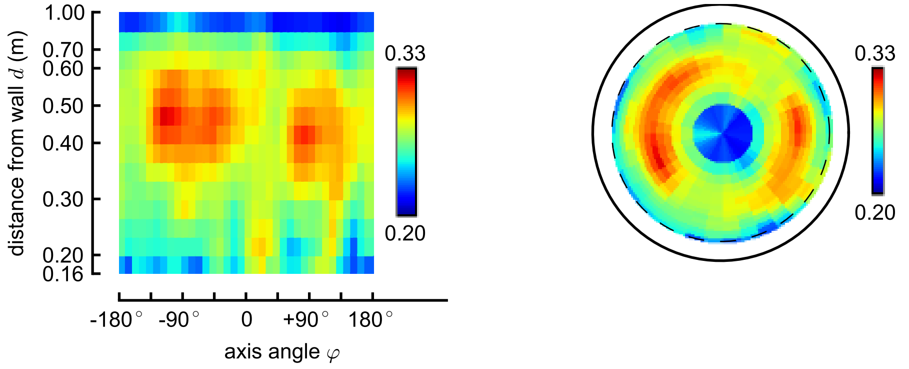

FIGURE 9.3. Number of detected saccades (both left and right)

(A) φ, d space (B) x^a, y^a space

Number of saccades

n^k

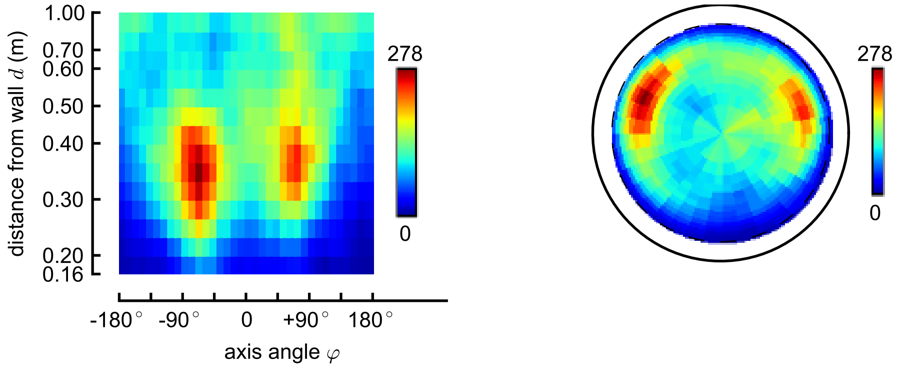

FIGURE 9.4. Number of detected left saccades

(A) φ, d space (B) x^a, y^a space

Number of left saccades

n_L^k

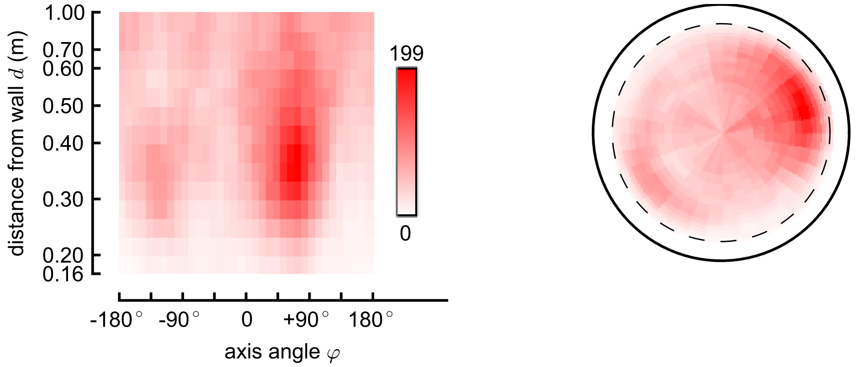

FIGURE 9.5. Number of detected right saccades

(A) φ, d space (B) x^a, y^a space

Number of right saccades

n_R^k

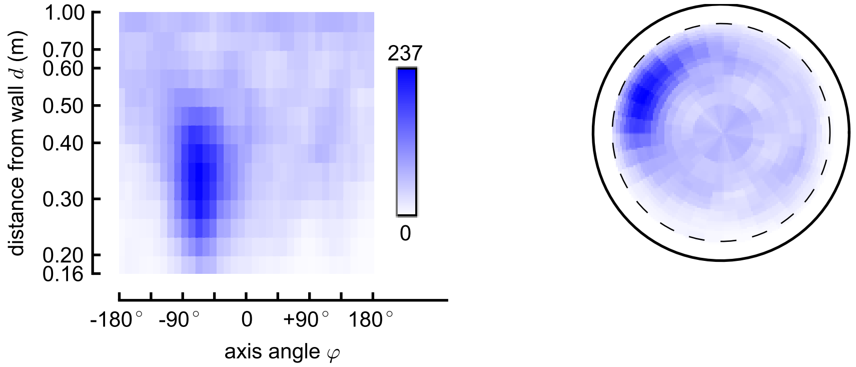

FIGURE 9.6. Observed saccade rate (both left and right)

(A) φ, d space (B) x^a, y^a space

Observed saccade rate

(m^k)

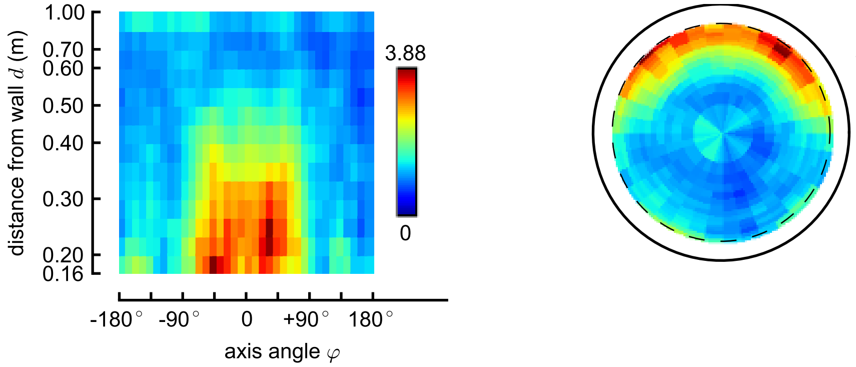

FIGURE 9.7. Observed left saccade rate

Observed left saccade rate

(m_L^k) saccades/s

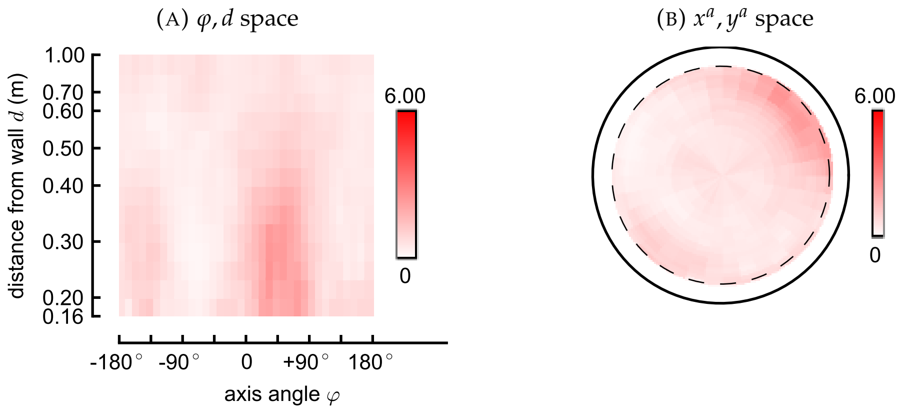

FIGURE 9.8. Observed right saccade rate

Observed right saccade rate

(m_R^k) saccades/s

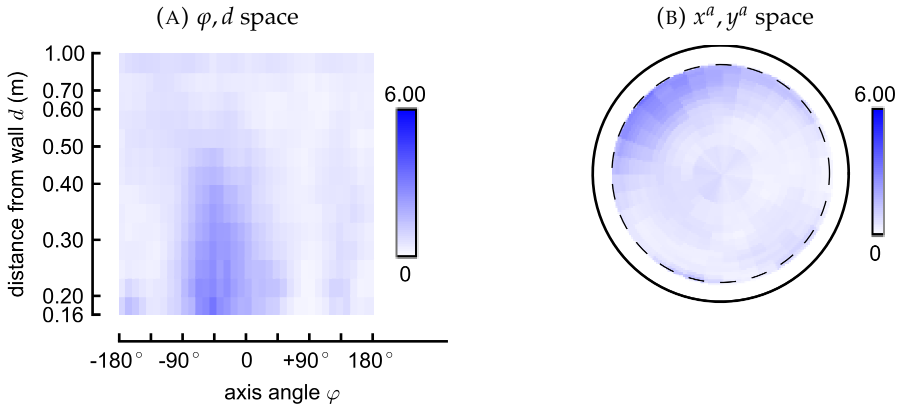

FIGURE 9.9. Observed left saccade rate

Estimated left saccade rate generation rate

r_L^k saccades/s

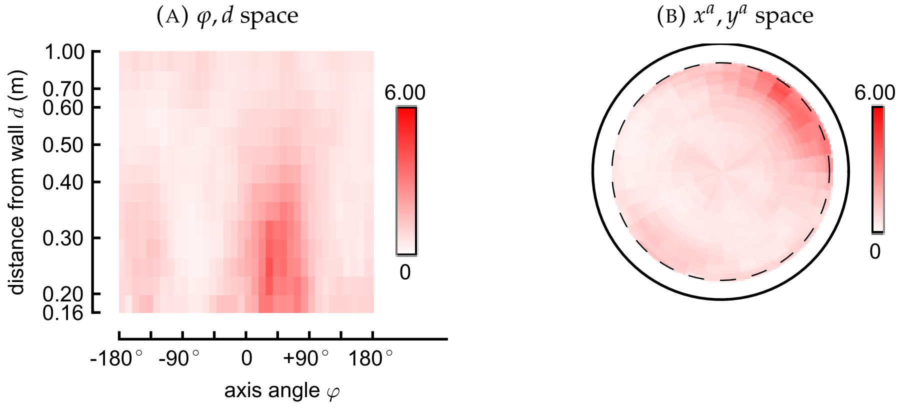

FIGURE 9.10. Observed right saccade rate

Estimated left saccade rate generation rate

r_R^k saccades/s

(A) φ, d space (B) x^a, y^a space

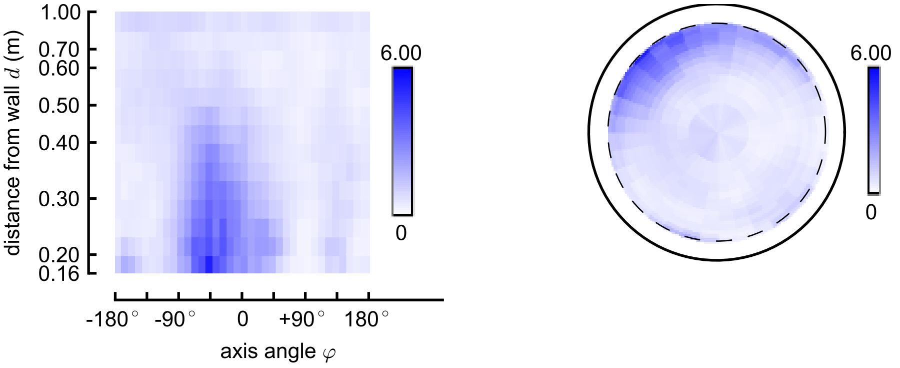

FIGURE 9.11. Order of left saccade rate

$\text{order}^k(\boldsymbol{r}_L)$

(A) φ, d space (B) x^a, y^a space

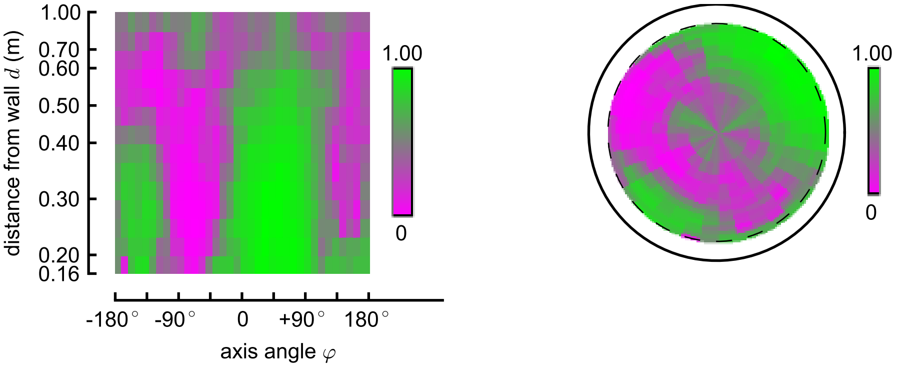

FIGURE 9.12. Order of right saccade rate

$\text{order}^k(\boldsymbol{r}_R)$

(A) φ, d space (B) x^a, y^a space

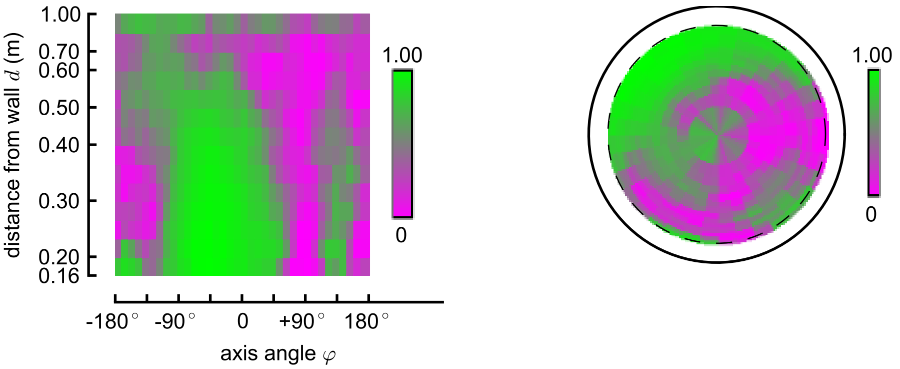

FIGURE 9.13. Estimated feature

(A) φ, d space (B) x^a, y^a space

Estimated feature

z^k

(arbitrary units)

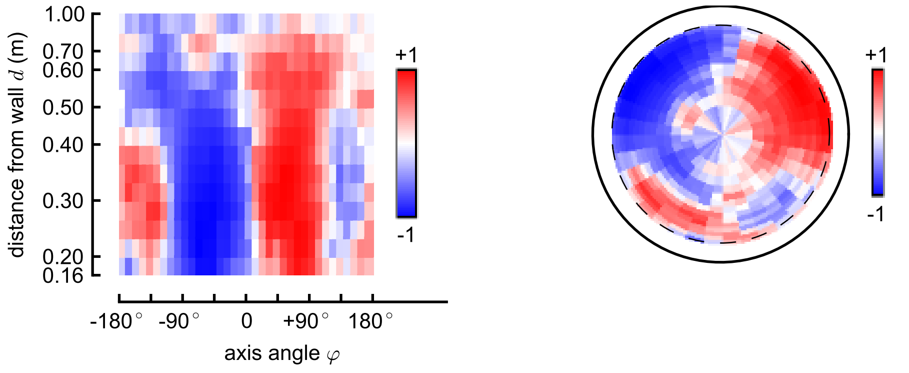

FIGURE 9.14. Uncertainty of estimated feature

(A) φ, d space (B) x^a, y^a space

Estimated feature

3std-dev(z^k)

(fraction)

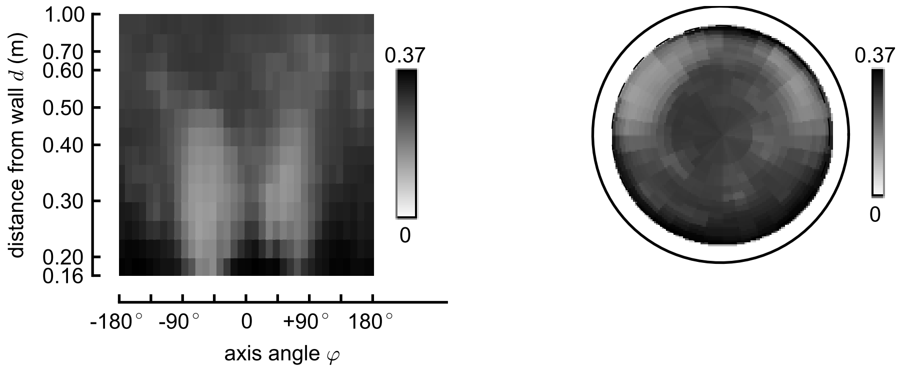

FIGURE 9.15. Observed saccade rates as a function of the estimated feature

(A) Mean (B) Confidence intervals

Observed event rates as a function of the estimated feature

m^k vs z^k

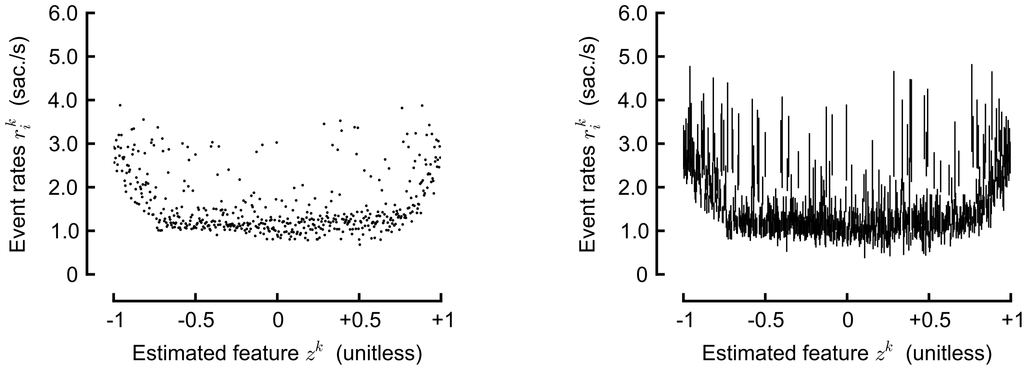

FIGURE 9.16. Observed saccade rates as a function of the estimated feature

(A) Mean — (B) Confidence intervals

Observed event rates as a function of the estimated feature

m_i^k vs z^k

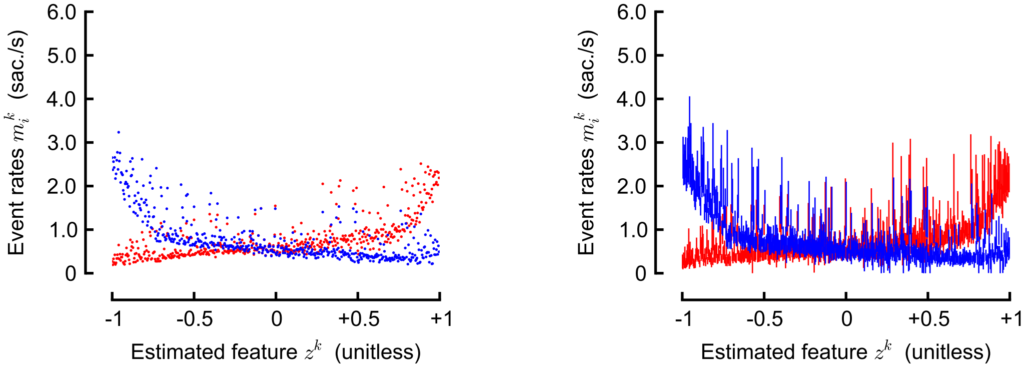

FIGURE 9.17. Estimated event generation rates as a function of the estimated feature

(A) Mean — (B) Confidence intervals

Event generation rates as a function of the estimated feature

r_i^k vs z^k

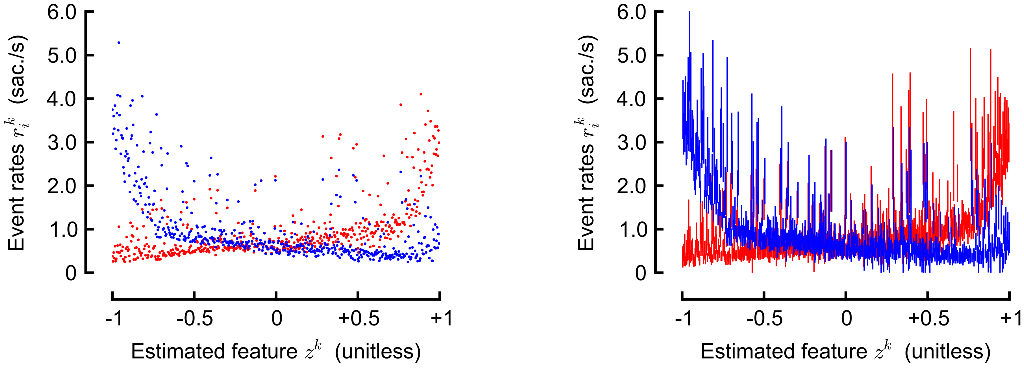

10. Complete plots (Angular-velocity based detector)

The next pages show the complete statistics using the events detected by the AVSD algorithm.

FIGURE 10.1. Number of detected saccades (both left and right)

Number of saccades

n^k

(A) φ, d space — (B) x^a, y^a space

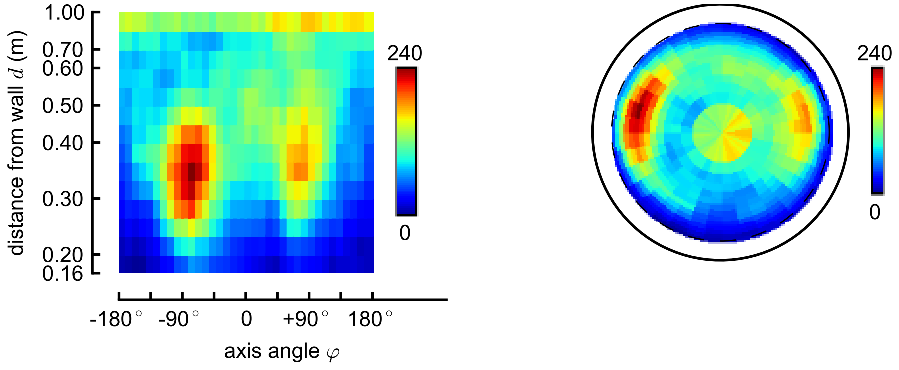

FIGURE 10.2. Number of detected left saccades

Number of left saccades

n^k_L

(A) φ, d space — (B) x^a, y^a space

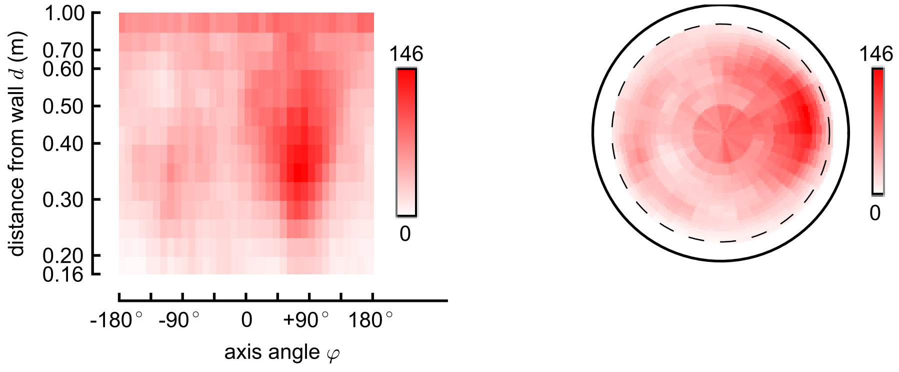

FIGURE 10.3. Number of detected right saccades

Number of right saccades

n^k_R

(A) φ, d space — (B) x^a, y^a space

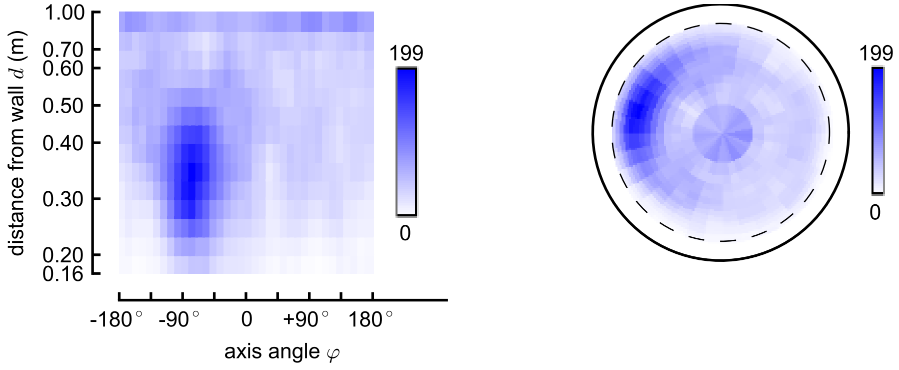

FIGURE 10.4. Observed saccade rate (both left and right)

Observed saccade rate

(m^k)

(A) φ, d space (B) x^a, y^a space

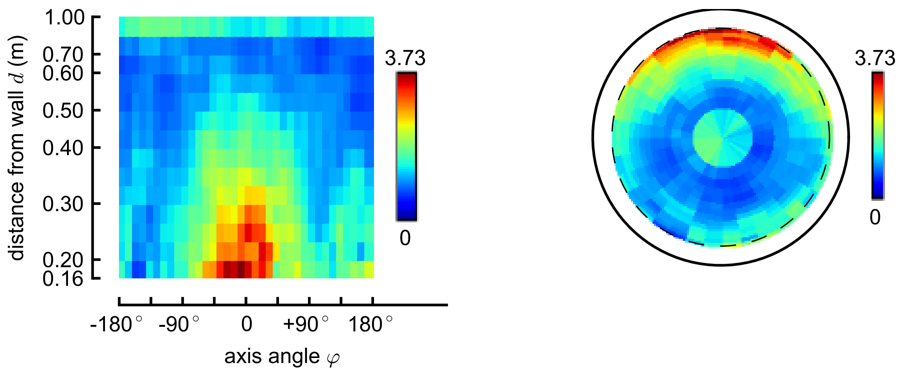

FIGURE 10.5. Observed left saccade rate

Observed left saccade rate

(m^k_L)

saccades/s

(A) φ, d space (B) x^a, y^a space

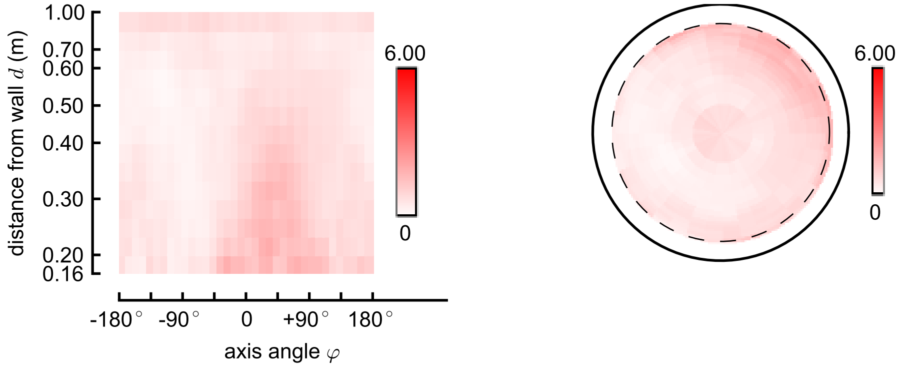

FIGURE 10.6. Observed right saccade rate

Observed right saccade rate

(m^k_R)

saccades/s

(A) φ, d space (B) x^a, y^a space

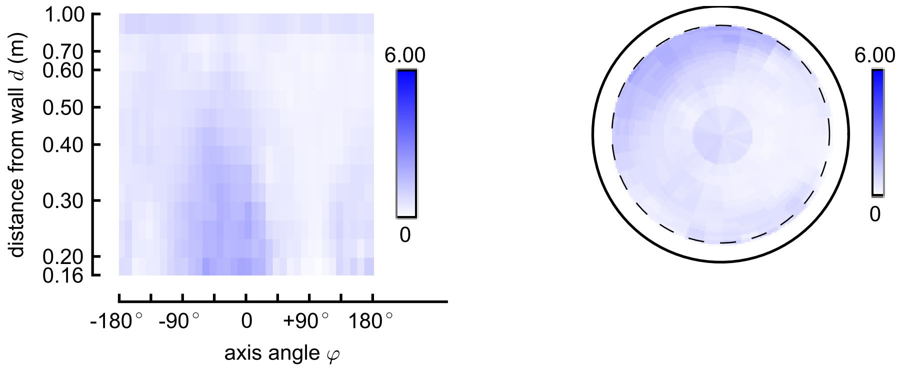

FIGURE 10.7. Observed left saccade rate

(A) φ, d space (B) x^a, y^a space

Estimated left saccade rate generation rate

r_L^k saccades/s

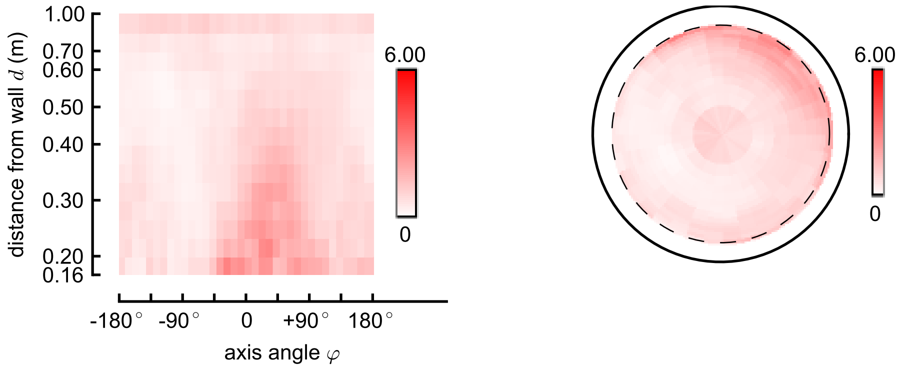

FIGURE 10.8. Observed right saccade rate

(A) φ, d space (B) x^a, y^a space

Estimated left saccade rate generation rate

r_R^k saccades/s

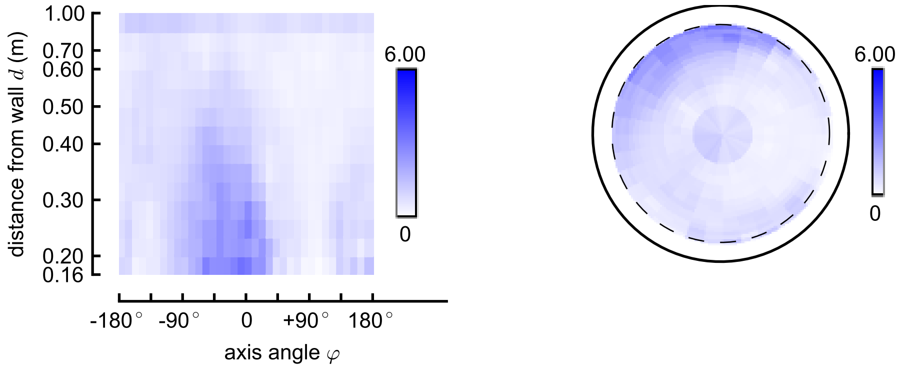

FIGURE 10.9. Order of left saccade rate

(A) φ, d space (B) x^a, y^a space

$\text{order}^k(\boldsymbol{r}_L)$

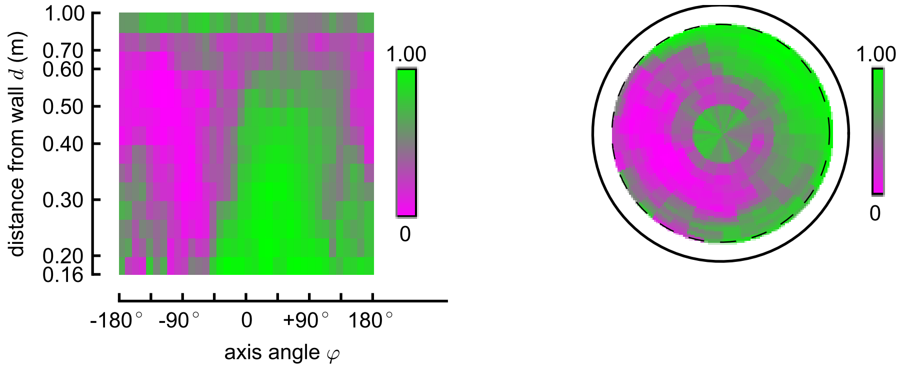

FIGURE 10.10. Order of right saccade rate

(A) φ, d space (B) x^a, y^a space

order$^k(\boldsymbol{r}_R)$

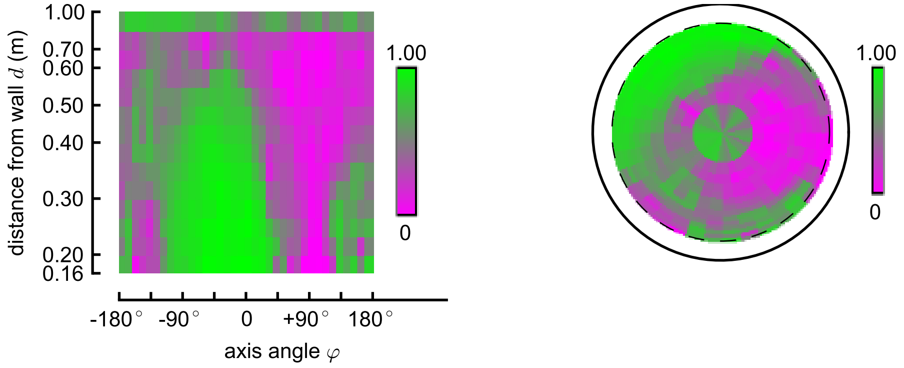

FIGURE 10.11. Estimated feature

(A) φ, d space (B) x^a, y^a space

Estimated feature

z^k (arbitrary units)

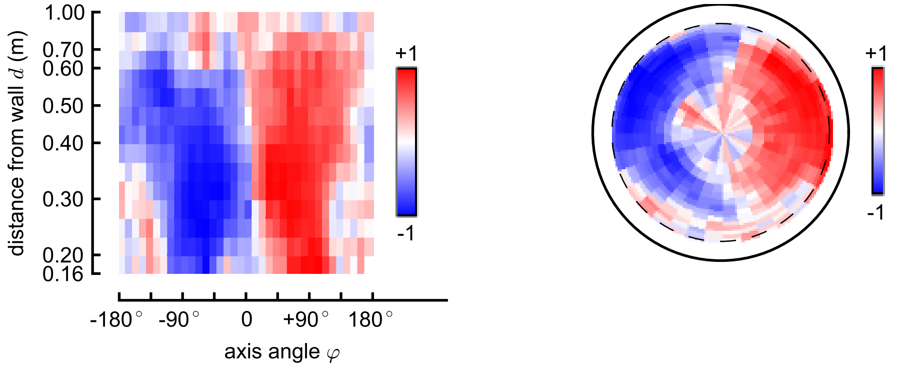

FIGURE 10.12. Uncertainty of estimated feature

(A) φ, d space (B) x^a, y^a space

Estimated feature

3std-dev(z^k) (fraction)

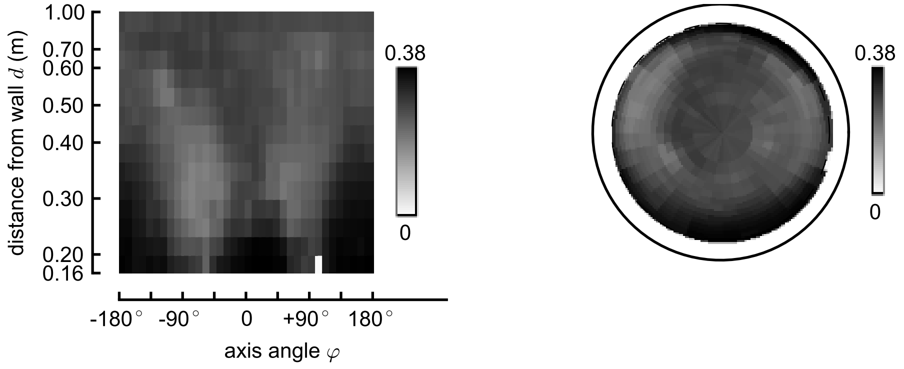

FIGURE 10.13. Observed saccade rates as a function of the estimated feature

Observed event rates as a function of the estimated feature

m^k vs z^k

(A) Mean | (B) Confidence intervals

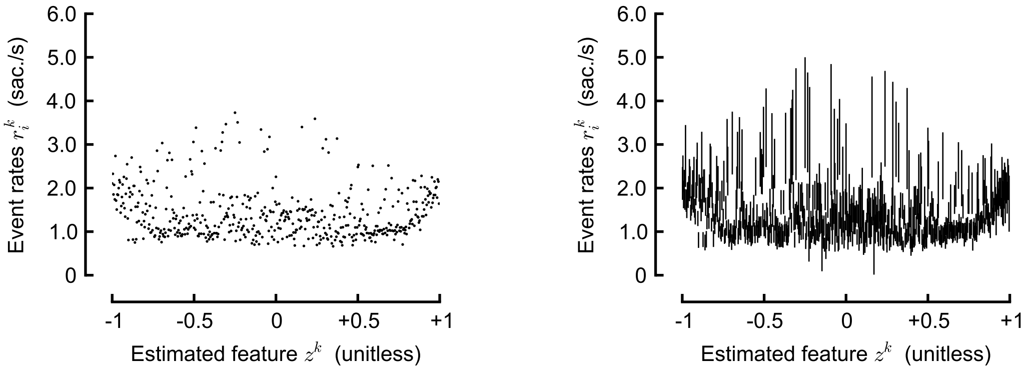

FIGURE 10.14. Observed saccade rates as a function of the estimated feature

Observed event rates as a function of the estimated feature

m_i^k vs z^k

(A) Mean | (B) Confidence intervals

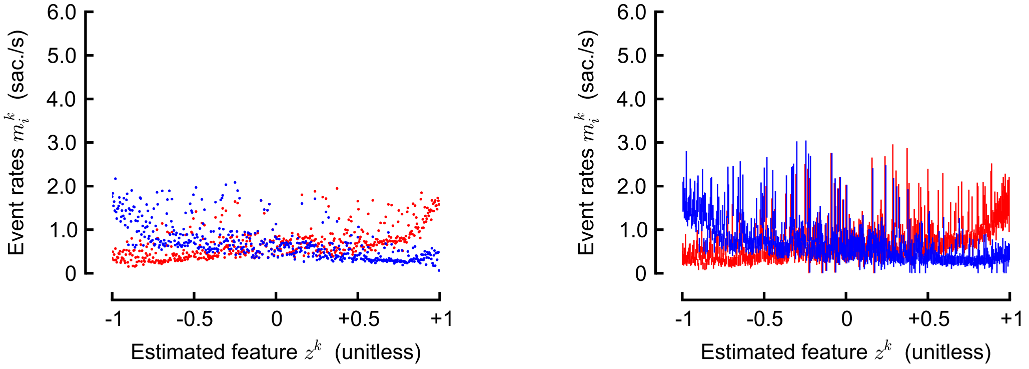

FIGURE 10.15. Estimated event generation rates as a function of the estimated feature

Event generation rates as a function of the estimated feature

r_i^k vs z^k

(A) Mean | (B) Confidence intervals

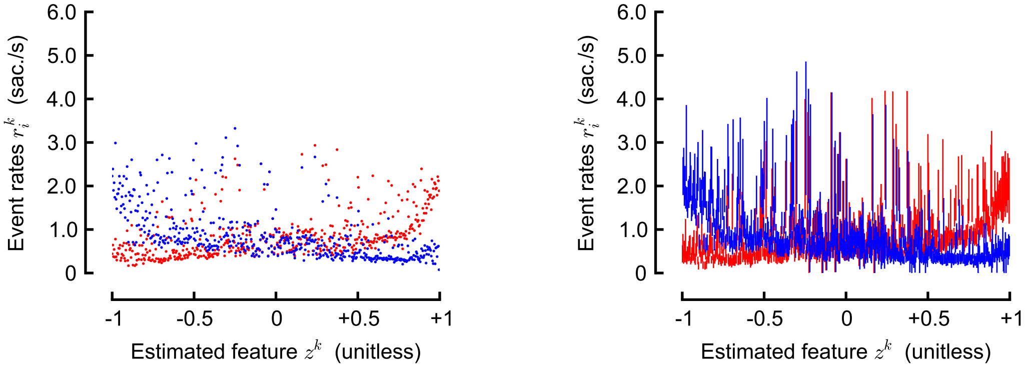

REFERENCES

[1] D. R. Cox and V Isham. *Point Processes*. Chapman & Hall, 1980. ISBN: 0-412-21910-7.

[2] V. Guerriero, A. Iannace, S. Mazzoli, M. Parente, S. Vitale, and M. Giorgioni. "Quantifying uncertainties in multi-scale studies of fractured reservoir analogues: Implemented statistical analysis of scan line data from carbonate rocks". In: *Journal of Structural Geology* 32.9 (2010), pp. 1271 –1278. ISSN: 0191-8141 DOI.
